# Molecular and iridescent feather reflectance data reveal recent genetic diversification and phenotypic differentiation in a cloud forest hummingbird

**DOI:** 10.1002/ece3.1950

**Published:** 2016-01-22

**Authors:** Juan Francisco Ornelas, Clementina González, Blanca E. Hernández‐Baños, Jaime García‐Moreno

**Affiliations:** ^1^ Departamento de Biología EvolutivaInstituto de EcologíaAC (INECOL)XalapaVeracruz91070Mexico; ^2^ Instituto de Investigaciones sobre los Recursos NaturalesUniversidad Michoacana de San Nicolás de HidalgoMoreliaMichoacánMexico; ^3^ Museo de ZoologíaDepartamento de Biología EvolutivaFacultad de CienciasUniversidad Nacional Autónoma de MéxicoMéxicoDF04510Mexico; ^4^ EsiLi ConsultancyHet Haam 166846 KWArnhemThe Netherlands

**Keywords:** Feather iridescence, glacial cycles, *Lampornis amethystinus*, Mesoamerican highlands

## Abstract

The present day distribution and spatial genetic diversity of Mesoamerican biota reflects a long history of responses to habitat change. The hummingbird *Lampornis amethystinus* is distributed in northern Mesoamerica, with geographically disjunct populations. Based on sampling across the species range using mitochondrial DNA (mtDNA) sequences and nuclear microsatellites jointly analysed with phenotypic and climatic data, we (1) test whether the fragmented distribution is correlated with main evolutionary lineages, (2) assess body size and plumage color differentiation of populations in geographic isolation, and (3) evaluate a set of divergence scenarios and demographic patterns of the hummingbird populations. Analysis of genetic variation revealed four main groups: blue‐throated populations (Sierra Madre del Sur); two groups of amethyst‐throated populations (Trans‐Mexican Volcanic Belt and Sierra Madre Oriental); and populations east of the Isthmus of Tehuantepec (IT) with males showing an amethyst throat. The most basal split is estimated to have originated in the Pleistocene, 2.39–0.57 million years ago (MYA), and corresponded to groups of populations separated by the IT. However, the estimated recent divergence time between blue‐ and amethyst‐throated populations does not correspond to the 2‐MY needed to be in isolation for substantial plumage divergence, likely because structurally iridescent colors are more malleable than others. Results of species distribution modeling and Approximate Bayesian Computation analysis fit a model of lineage divergence west of the Isthmus after the Last Glacial Maximum (LGM), and that the species’ suitable habitat was disjunct during past and current conditions. These results challenge the generality of the contraction/expansion glacial model to cloud forest‐interior species and urges management of cloud forest, a highly vulnerable ecosystem to climate change and currently facing destruction, to prevent further loss of genetic diversity or extinction.

## Introduction

Geographic isolation is the most widely accepted mode of speciation by which populations differentiate (Coyne and Orr 2004). In this mode, populations across the species’ range become dissected into two groups by a physical barrier that prevents gene flow between them. With allopatry induced by the physical isolation of populations interrupting gene flow between allopatric sister populations isolated by geographic barriers, genetic divergence accrues as a result of adaptation to the prevailing environmental conditions and by means of genetic drift (Coyne and Orr 2004) and the allopatric sister populations will differentiate phenotypically given sufficient time or selection pressures (Coyne and Orr 2004; Nosil 2008a; Price 2008; Winger and Bates 2015). Migration and gene flow may occur between populations if geographical barriers are permeable (e.g., Rodríguez‐Gómez et al. 2013; Rodríguez‐Gómez and Ornelas 2015), but despite high levels of gene flow, phenotypic plasticity and/or selection may be strong enough to the maintenance of phenotypic divergence (Jordan et al. 2005; Niemiller et al. 2008; Nosil 2008a; Milá et al. 2009; González and Ornelas 2014). In the ‘divergence with gene flow’ model (Endler 1977), the physical barrier to gene flow is absent or the continuous divergence process leading to speciation is incomplete (Nosil 2008b). Thus, a coalescent‐based analysis is needed to distinguish the effects of time of isolation and gene flow on levels of genetic divergence using the ‘isolation with migration’ model (Hey 2006). Although geographical isolation leading to genetic divergence is traditionally considered fundamental to phenotypic divergence (Mayr 1963), empirical work suggests that strong selection can still lead to both genetic and phenotypic divergence in spite of high levels of gene flow (e.g., Smith et al. 1997; Jordan et al. 2005; Parra 2010; González and Ornelas 2014).

**Figure 1:**
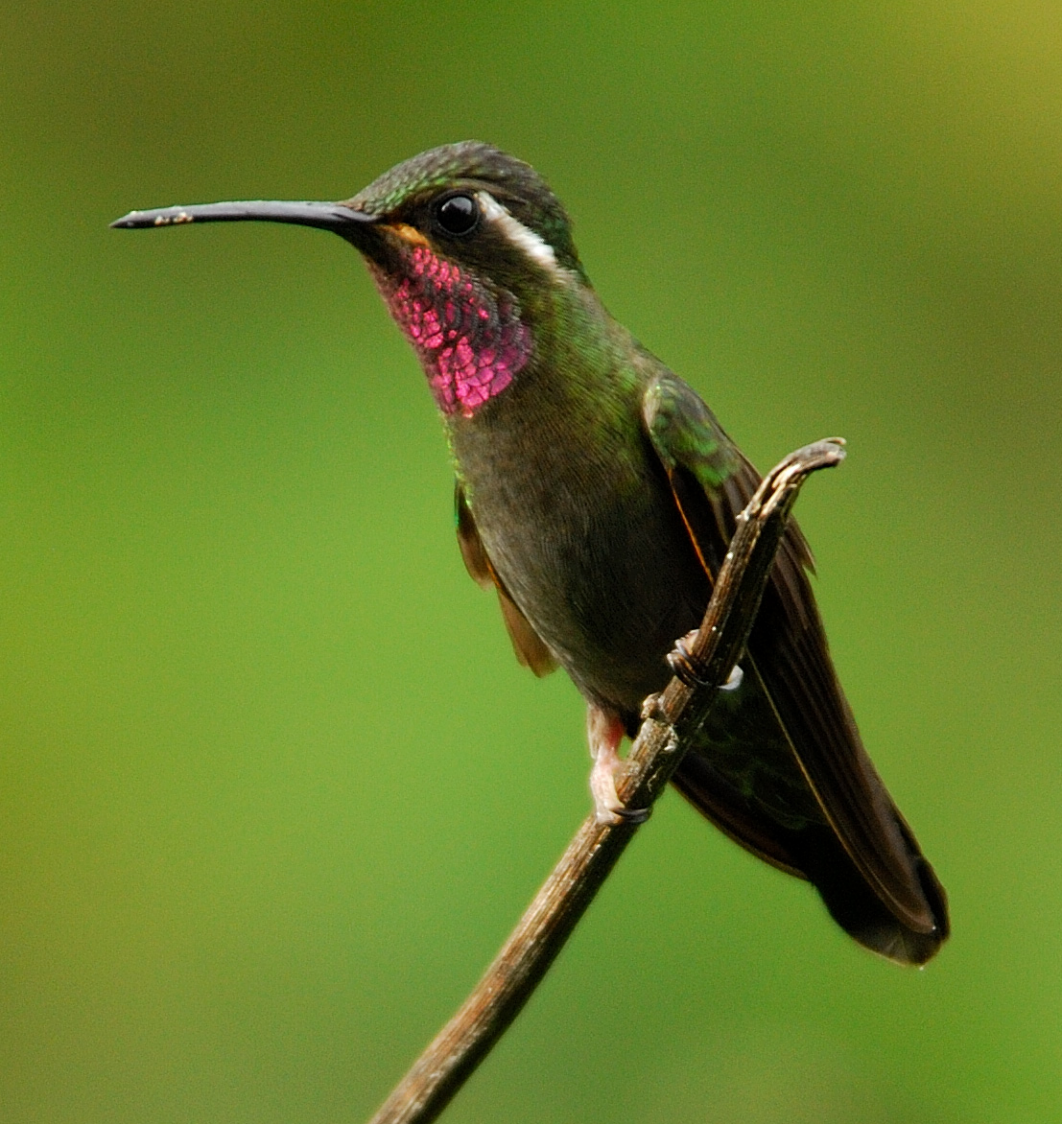
A male amethyst‐throated hummingbird (*Lampornis amethystinus*). Photograph by Knut Eisermann.

Here, the phylogeography of the cloud forest‐interior amethyst‐throated hummingbird, *Lampornis amethystinus* (Swainson, 1827) (Trochilidae), is examined through phylogeographic and population genetic analyses of nuclear and mitochondrial DNA data. *Lampornis amethystinus* is a resident hummingbird species to the cloud forest in the Mesoamerican highlands (Fig. 1), with complex vocalizations and aerial displays (Ornelas et al. 2002) and disjunct distribution spanning the Trans‐Mexican Volcanic Belt (TMVB), Sierra Madre Oriental, Sierra de Los Tuxtlas, and Sierra Madre del Sur to the interior highlands of Chiapas, Guatemala, El Salvador, and Honduras (Howell and Webb 1995). The species is composed of two groups: the widespread *amethystinus* with males showing gorgets glittering rose‐pink (amethyst), and *margaritae* restricted from Michoacán to Oaxaca (possibly sympatric with *amethystinus* in southern Oaxaca) with gorgets glittering bluish violet (Howell and Webb 1995). Dickinson (2003) recognized six subspecies based on distribution and geographic variation in size and plumage coloration: *amethystinus* (Sierra Madre Oriental, Sierra de Los Tuxtlas and eastern TMVB), *brevirostris* (western TMVB), *margaritae* (Guerrero), *circumventus* (Sierra de Miahuatlán, Oaxaca), *salvini* (Chiapas, Guatemala and El Salvador), and *nobilis* (central Honduras) (see Fig. 1 of Cortés‐Rodríguez et al. 2008). However, there is no agreement with regard the phenotypic differences distinguishing these subspecies and their taxonomic status (AOU 1998). Cortés‐Rodríguez et al. (2008) used mtDNA sequences to derive a first perspective on the evolutionary history of *L. amethystinus* populations, focusing on genetic differentiation and geographic variation in gorget color. Two haplogroups separated by the IT were identified but the existence of other mtDNA lineages corresponding to gorget‐color differences was not supported. Using the Cortés‐Rodríguez et al. (2008) dataset, Barber and Klicka (2010) estimated that the split between populations separated by the Isthmus occurred ca. 1 MYA and, with the addition of new samples, Ornelas et al. (2013) estimated the split at 1.07 MYA (95% HPD 1.46–0.71 MYA). Based on the observed mtDNA differences between haplogroups, Cortés‐Rodríguez et al. (2008) proposed species recognition as originally proposed by Ridgway (1911): *L. amethystinus* for populations west of the isthmus, and *L. salvini* to eastern populations, but their taxonomic proposal should await study with additional nuclear markers to produce more accurate and precise estimates of divergence, particularly in light of the low Bayesian posterior probabilities associated with the proposed split.

Here, we used a multilocus approach and a broad geographical sampling of *L. amethystinus* to test whether the blue‐throated form (*margaritae*) found exclusively in the Sierra Madre del Sur evolved recently from other amethyst‐throated lineages distributed in adjacent mountain ranges or instead the blue‐throated form resulted form more complex population divergence scenarios and long‐term isolation. Patterns of morphological, genetic diversity (mtDNA and nuclear microsatellites), haplotype genealogy, and genetic structure were examined to infer the distributional, demographic, and evolutionary history of the amethyst‐throated hummingbird. In addition, we estimated the timing of divergence and gene flow rates between the blue and amethyst lineages to compare the evolution of phenotypic variation with that of major climatic events during the Quaternary. To frame the information derived from genetic analyses in an explicitly paleoecological context, we constructed species distribution models to predict where populations of amethyst‐throated hummingbirds resided during the LGM and Last Interglacial (LIG) climate conditions, and whether populations were connected during those events. The distribution and composition of the Mesoamerican biota have been strongly influenced by geological and climatic events, with considerable invasions of Mesoamerica by South American tropical elements and temperate elements from North America prior to the formation of the Isthmus of Panama ca. 4.5 MYA (Ornelas et al. 2010, 2014; Ruiz‐Sanchez and Ornelas 2014), and forest fragmentation with species restricted to refuge populations in Mesoamerica during glacial maxima of the Pleistocene (1.6–0.01 MYA) (Hewitt 2000). Recent studies have explored the phylogeography of widespread, cloud forest‐adapted species across northern Mesoamerica. Some studies found that the genetic divergence in the region was shaped by the repeated cycles of cloud forest contraction and expansion due to Pleistocene climatic cycling, producing either a phylogeographical break at the Isthmus of Tehuantepec (e.g., González et al. 2011; Gutiérrez‐Rodríguez et al. 2011; Rodríguez‐Gómez et al. 2013; Ornelas and Rodríguez‐Gómez 2015), whereas others revealed stronger signals of isolation and genetic structure associated with historical cloud forest fragmentation and mountain geography (Ornelas and González 2014). Nonetheless, comparative tests of simultaneous diversification revealed that the observed phylogeographical breaks in the region occurred as multiple vicariant events at different times (Ornelas et al. 2013). In addition to addressing the evolutionary history of amethyst‐throated hummingbirds and phenotypic divergence in gorget coloration, our results can shed some light on how populations of cloud forest‐adapted species in northern Mesoamerica responded to climate change during the Pleistocene glacial cycles.

## Materials and Methods

### Sampling

To supplement the mtDNA dataset in Cortés‐Rodríguez et al. (2008) (*n *=* *69 individuals), we present new sequence data for 88 additional individuals (Table S1). The 157 individuals were sampled from 43 localities and categorized into five groups based on mountain geography: SMO* *=* *Sierra Madre Oriental; TUX* *=* *Sierra de Los Tuxtlas and Sierra de Santa Marta; SMS* *=* *Sierra Madre del Sur (Guerrero and Sierra de Miahuatlán, Oaxaca); TMVB* *=* *Trans‐Mexican Volcanic Belt; CHIS* *=* *Chiapan Highlands separated by the Central Depression that together with Guatemala and El Salvador form the Trans‐Isthmian Highlands region (TIH); Fig. S1 and Table S1). The sampling presented in this study practically covers the entire distribution of amethyst‐throated hummingbirds in Mexico. Birds were captured in mist nets and two rectrices or tissue samples were collected for subsequent genetic analysis. Samples were collected using required permits and approved animal welfare protocols. We also obtained sequence data or downloaded sequences from GenBank for congeners (*L. clemenciae*,* L. viridipallens*,* L. hemileucus*,* L. calolaemus, L. sybillae*), *Lamprolaima rhami*,* Doricha eliza*,* Calothorax pulcher*,* Selasphorus platycercus,* and *Archilochus colubris* to be used as outgroups according to García‐Moreno et al. (2006), McGuire et al. (2014) and Ornelas et al. (2014).

### Mitochondrial DNA sequencing and microsatellite genotyping

Two mitochondrial genes—349 base pairs (bp) of NADH nicotinamide dehydrogenase subunit 2 (*ND2*) and 402 bp of cytochrome *b* (cyt *b*) genes were amplified by PCR and sequenced to infer phylogenetic relationships among haplotypes. Genomic DNA was extracted using Chelex (10%) or the extraction DNeasy blood and tissue kit (Qiagen, Valencia, CA), following the protocol recommended by the manufacturer. Amplification of *ND2* was conducted with primers L5215–H5578 (Hackett 1996), whereas for the cyt *b* we used L15560–H16064 (Sorenson et al. 1999). The 14 *μ*L PCR mix for both fragments contained a final concentration of 0.72 × PCR buffer (Promega, Madison, WI), 3.6 mmol/L^−1^ MgCl_2_, 0.58 mmol/L^−1^ dNTPs, 0.4 *μ*g/*μ*L BSA, 0.18 *μ*mol/L^−1^ of each primer, 0.04 U Taq polymerase (Promega), and 1–1.5 *μ*L of genomic DNA. The PCR cycling conditions for *ND2* consisted of an initial denaturation at 95°C for 2 min, followed by 35 cycles of denaturation at 95°C for 20 sec, annealing at 47°C for 20 sec and an extension of 74°C for 1 min, and a final extension of 72°C for 3 min. For the cyt *b* the PCR cycling conditions consisted of an initial denaturation at 80°C for 5 min, followed by 35 cycles consisting of denaturation at 95°C for 1 min, and annealing at 47°C for 4 min, and a final extension of 66°C for 10 min. PCR products visualized on 1% agarose gels stained with ethidium bromide were purified with the QIAquick kit (Qiagen, Inc.) and sequenced using the BigDye Terminator Cycle Sequencing kit (Applied Biosystems, Ann Arbor, MI). Sequences were read in a 310 automated DNA sequencer (Applied Biosystems, Carlsbad, CA) at the INECOL's sequencing facility. Finally, sequences were assembled using Sequencher ver. 5.2.3 (Gene Codes, Ann Arbor, MI) and then manually aligned with SE‐AL ver. 2.0a11 (http://evolve.zoo.ox.ac.uk/software.html). All newly acquired sequences have been deposited in GenBank (Accession nos. KU375264–KU375338, KU375339–KU375423).

Samples from 126 hummingbirds were genotyped at eight autosomal microsatellite loci designed for *Campylopterus curvipennis* (Molecular Ecology Resources Primer Development Consortium et al. 2010; GenBank accession nos. GQ294539–GQ294550) and *Selasphorus platycercus* (Oyler‐McCance et al. 2011; HQ316946–HQ316955). Amplification of microsatellite loci were performed with the Multiplex PCR kit (Qiagen) using two mixes of four fluorescently labelled primers (Applied Biosystems). Multiplex PCR amplification (5 *μ*L total volume) contained final concentrations of 1× Multiplex PCR Master Mix, 0.2 *μ*mol/L^−1^ of primer mix, and 0.5 *μ*L of DNA. Alleles visualization and fragment sizing were performed using GENEMAPPER ver. 3.2 (Applied Biosystems) against an internal size standard (GeneScan‐600LIZ; Applied Biosystems) and scored manually. A full description of the development protocol for the loci can be found at the Molecular Ecology Resources Database (http://tomato.biol.trinity.edu/; Molecular Ecology Resources Primer Development Consortium et al. 2010) for Cacu16‐1 and Cacu17‐2 and Oyler‐McCance et al. (2011) for HumB2, HumB3, HumB9, HumB10, HumB11, and HumB15.

### Relationships among haplotypes

To infer genealogical relationships among haplotypes, a statistical parsimony network for the combined mtDNA dataset was constructed as implemented in TCS ver. 1.2.1 (Clement et al. 2000), with the 95% connection probability limit and treating gaps as single evolutionary events. Loops were resolved following the criteria given by Pfenninger and Posada (2002).

### Genetic diversity and population structure

#### Analysis of mtDNA sequence data

Haplotype diversity (*h*) and nucleotide diversity (*π*) for each geographical group, and pairwise comparisons of *F*_ST_ values between populations and groups with 1000 permutations were calculated using ARLEQUIN ver. 3.5 (Excoffier and Lischer 2010). Note that ‘populations’ are sampling localities (*n *=* *43), whereas ‘groups’ are sets of pooled populations (*n *=* *5), as specified in Table S1. To determine whether or not populations are geographically structured, three analyses of molecular variance (AMOVAs; Excoffier et al. 1992) were run based on pairwise differences using ARLEQUIN with populations (1) treated as a single group to determine the amount of variation partitioned among and within locations, and (2) grouped into east and west of the IT or (3) grouped into five areas based on mountain geography (SMO, TUX, TMVB, SMS, CHIS; Fig. S1 and Table S1). AMOVAs were run using the Tamura and Nei model with 10,000 permutations to determine the significance of each AMOVA using the combined *ND2 *+* *cyt *b* dataset.

#### Analysis of microsatellite data

Expected and observed heterozygosity, mean number of alleles per locus in each population, the extent of linkage disequilibrium between pairs of loci, and departures from Hardy‐Weinberg equilibrium (HWE) within populations and loci were calculated using GENEPOP ver. 3.4 (Raymond and Rousset 1995), with Bonferroni correction applied to correct for multiple simultaneous comparisons. In addition, allelic richness, a measure of the number of alleles per locus among populations independent of the sample size, was calculated in FSTAT ver. 2.9.3 (Goudet 1995). Null allele frequencies for each locus were estimated using MICRO‐CHECKER ver. 2.23 (Van Oosterhout et al. 2004).

To investigate population genetic structure, we calculated global and pairwise comparisons of *F*_*ST*_ values between populations using FSTAT with 10,000 permutations. *F*_*ST*_ estimates perform better than *R*_*ST*_ when sample sizes are small and the number of loci scored is low (Gaggiotti et al. 1999). In addition, patterns of genetic structure for microsatellites were evaluated using the Bayesian Markov chain Monte Carlo (MCMC) clustering analysis in STRUCTURE ver. 2.1 (Pritchard et al. 2000). We ran STRUCTURE under the admixture model with correlated allele frequencies and the LOCPRIOR function (Pritchard et al. 2000). Twenty independent chains were run for each *K*, from *K *=* *1 to *K *=* *7. The length of the burn‐in was 500,000 and the number of MCMC replications after the burn‐in was 1,000,000. The most likely number of populations was evaluated by calculating Δ*K* values (Evanno et al. 2005).

### Demographic history

The demographic history of each *L. amethystinus* group (Fig. S1) was inferred by means of neutrality tests and mismatch distributions constructed in ARLEQUIN. To test whether populations evolved under neutrality, Fu's *Fs* test and Tajima's *D* tests were calculated with 1000 permutations, and mismatch distributions were calculated using the sudden expansion model of Schneider and Excoffier (1999) with 9000 bootstrap replicates. The validity of the sudden expansion assumption was determined using the sum of squared deviations (SSD) and Harpending's raggedness index (Hri), which are higher in stable, nonexpanding populations (Rogers and Harpending 1992). We also used Bayesian skyline plots (BSP; Drummond et al. 2005) performed in BEAST ver. 1.6.1 (Drummond and Rambaut 2007) for mtDNA to assess temporal variation in effective population size (*N*_*e*_). This analysis was performed for each of the geographic groups separately based on BEAST and AMOVA results with the same settings used in BEAST for divergence time estimation (see below), except that the coalescent tree prior was specified as Bayesian skyline with five groups. Three runs of 10 million steps and effective sample sizes (ESS) > 200 were compared to ensure convergence. Outputs were combined in LOGCOMBINER ver. 1.6.1 (Drummond and Rambaut 2007) and visualized in TRACER ver. 1.6.0 (http://tree.bio.ed.ac.uk/software/tracer/). The time axis was scaled using the geometric mean substitution rate of 0.010075 substitutions per site per lineage per million years (s/s/l/MY), according to the average rates of 0.029 s/s/MY for *ND2* and 0.014 s/s/MY for cyt *b* obtained for Hawaiian honeycreepers (Lerner et al. 2011).

### Divergence time estimation

We estimated divergence times among groups with the Bayesian approach implemented in BEAST using the combined mtDNA (*ND2* and cyt *b*) sequences and the closest nucleotide substitution model under the Bayesian information criterion (BIC), GTR, suggested by jMODELTEST ver. 0.1.1 (Posada 2008) as the clock model. The ingroup comprised all newly acquired mtDNA sequences of *L. amethystinus* and *ND2* and cyt *b* sequences downloaded from GenBank of Cortés‐Rodríguez et al. (2008), and *Lampornis clemenciae*,* L. sybillae*,* L. viridipallens*,* L. calolaemus*,* Lamprolaima rhami*,* Doricha eliza*,* Calothorax pulcher*,* Selasphorus platycercus,* and *Archilochus colubris* of García‐Moreno et al. (2006), McGuire et al. (2014) and Ornelas et al. (2014) used as multiple outgroups. The bee hummingbird group, mountain gems, and *L. amethystinus* were constrained to be monophyletic based on McGuire et al. (2014) and Ornelas et al. (2014). We ran BEAST two times for 10 million generations, sampling every 1000 steps and discarding the first 10% of trees as burn‐in, using a coalescent tree prior assuming constant population size, and the mitochondrial geometric mean substitution rate of 0.01075 s/s/l/MY obtained for Hawaiian honeycreepers (Lerner et al. 2011) to calibrate the tree. To calibrate the root, we used 12.8 MYA (normal prior, SD 2.0 MYA, range of 16.09–9.51 MYA; Smith and Klicka 2010) divergence time for the split between mountain gems and bee hummingbirds. The coalescent tree prior used in this analysis appears to be a better fit when datasets composed of both interspecific and intraspecific data are predominantly intraspecific (Ho et al. 2011). We combined log and trees files from each independent run using LOGCOMBINER, then viewed the combined log file in TRACER to ensure that ESS values for all priors and the posterior distribution were >200, and finally annotated the trees using TREEANNOTATOR ver. 1.6.1 (Drummond and Rambaut 2007) summarized as a maximum clade credibility tree with mean divergence times and 95% highest posterior density (HPD) intervals of age estimates and visualized in FIGTREE ver. 1.3.1 (http://tree.bio.ed.ac.uk/software/figtree/).

### Historical and contemporary gene flow

The isolation‐with‐migration (IM) coalescent model implemented in IMa (Hey and Nielsen 2004, 2007) was used to determine whether recent genetic divergence between groups of populations (see Results) occurred with gene flow. Several preliminary runs of IMa were conducted to optimize priors using mtDNA and microsatellite data to then estimate the effective population size of the ancestral (*q*_a_) and the two descendant populations (*q*_1_ and *q*_2_), effective number of migrants per generation in both directions (*m*_1‐to‐2_ and *m*_2‐to‐1_), and time since divergence (*t*) at which the ancestral population gave rise to the descendant populations. IM models search parameter space for the most likely estimates using a Bayesian framework assuming random mating within populations and that populations are each other's closest relatives not exchanging genes with other nonsampled populations (Hey and Nielsen 2004; Hey 2006). We used IMa on a subsample of 10–48 individuals from each population combining their microsatellite genotypes with 349 bp of mitochondrial *ND2* and 402 bp of cyt *b* sequences. The isolation‐with‐migration model implemented in IMa involves several simplifying assumptions, including no recombination within each locus, no population structure within each species, no genetic contribution from unsampled populations, and selective neutrality. Although we recognized that our data may violate some of the IM model assumptions, previous work has shown that IM models, as applied in IMa, are generally quite robust to small‐to‐moderate violations of the IM model assumptions (Strasburg and Rieseberg 2010). In particular, random mating within populations (panmixia) has little effect on parameter estimates ever for fairly high levels of population structure, and those involving small to moderate levels of introgression among considered taxa (Strasburg and Rieseberg 2010). Another important assumption of the IM model is that the populations in question have most recently split from one another. A violation of this assumption is possible because even moderate levels of gene flow from an unsampled third population may overestimate divergence times. However, we restricted our IM analyses to adjacent currently isolated populations that more likely have evolved under a divergence scenario in the face of gene flow.

Initial runs were conducted searching for suitable conditions to constrain parameter intervals and to alter the heating scheme to achieve sufficient mixing among chains (Hey and Nielsen 2007). The final runs were carried out with a Hasegawa‐Kishino‐Yano (HKY) mutation model (Hasegawa et al. 1985), a chain length of 2 million steps after a burn‐in of 1 million steps using 15 chains for the joint mtDNA and microsatellites dataset, and a geometric heating scheme using high values (g1 = 0.85 and g2 = 0.95). We present results from two independent runs that were conducted using identical conditions, but different starting points. We confirmed sufficient mixing by observing that ESS values were ≥50 and inspecting parameter plots for trends (Hey and Nielsen 2007). We used the geometric mean substitution rates of 3.99 × 10^−4^ substitutions per site per year (s/s/yr) for the ten loci according to the averages of 2.9 × 10^−8^ s/s/MY for *ND2* and 1.4 × 10^−8^ s/s/MY for cyt *b* obtained for Hawaiian honeycreepers (Lerner et al. 2011) and 1.08 × 10^−3^ s/s/yr based on an average mutation rate of 2.96 × 10^−3^ s/s/generation for microsatellites (Ortego et al. 2008), to estimate the effective population sizes of each genetic group. The mutation rate was converted to per locus rate by multiplying the fragment length in base pairs for conversion to demographic units (Hey and Nielsen 2007). Although there is considerable uncertainty in the determination of these rates, they have been applied here systematically to all coalescent‐based assessments. Consequently, estimates presented are relative to one another, and although not necessarily exact, they still likely reflect relative migration rates among populations. To convert the effective population size estimates, we used a 2.75‐years generation time which is the average of those proposed for other hummingbird species based on the observation that the age of maturity begins 1 year after hatching, and an assumed low annual adult survival rate of 0.3 reported for *Colibri thalassinus* (Ruiz‐Gutiérrez et al. 2012), *Augastes scutatus* (Da Cruz Rodrigues et al. 2013), and *Archilochus colubris* (Hilton and Miller 2003) or a high annual adult survival rate of 0.52 for an emerald resident species, *Hylocharis leucotis* (Ruiz‐Gutiérrez et al. 2012). The approximate average generation time (*T*) is calculated according to *T* = *a *+ [*s*/(1–*s*)] (Lande et al. 2003), where *a* is the time to maturity and *s* is the adult annual survival rate. Based on this, estimates for *T* range from 2.43 to 3.08 years (average 2.75 years). To convert time since divergence parameter of IMa to years, *t*, we divided the time parameter (*B*) by the mutation rate per year (*U*) converted to per locus rate by multiplying by the fragment length in base pairs.

### Analyses of population history with coalescence models

We infer the population history of amethyst‐throated hummingbirds using DIYABC ver. 2.0 (Cornuet et al. 2014), a coalescence‐based program that infers the population history by looking backwards in time to examine the genealogy of alleles until reaching the most recent common ancestor using approximate Bayesian computation algorithm (ABC) (Cornuet et al. 2008). Populations covering the whole species’ distribution were analysed to infer the history of the genetic structure indicated by STRUCTURE and BEAST analyses. Using the DIYABC software (Cornuet et al. 2014), we simulated and compared through posterior probabilities three simple population demography scenarios considering both mtDNA sequences and microsatellites and parameter prior distributions based on results of BEAST, BSP, and IMa analyses (see Results). The evolutionary scenarios were built considering the STRUCTURE and BEAST analyses, which point to an older divergence between CHIS and the rest of groups west of IT (SMS, SMO and TMVB), and different combinations of splitting of unresolved relationships among the SMS, SMO, and TMVB geographic groups. Individuals from the TUX population were not included due to the small sample size. The first scenario (Sc1, *isolation split model 1*) predicts that TMVB (Pop1) merged with SMO (Pop2) at t1 then SMO merged with SMS (Pop3, *margaritae*) at t2 and subsequently with CHIS east of IT (Pop4) at t3. This scenario was expected to be the most likely according with hierarchical STRUCTURE and BEAST analyses. The second scenario (Sc2, *isolation split model 2*) is similar to the previous one but predicts that SMS (Pop3) merged with TMVB (Pop1) at t1 then TMVB merged with SMO (Pop2) at t2 and subsequently with CHIS east of IT (Pop4) at t3. The third scenario (Sc3, *isolation with admixture model*) consisted of the same basal split between CHIS (Pop4) and the rest of groups west of IT described in previous scenarios but includes a hybridization/lineage fusion event in which SMS (Pop3) is the descendent of admixture between TMVB (Pop1) and SMO (Pop2) at t1, then Pop1 merged with Pop2 at t2, and subsequently with Pop4 at t3. Although there are numerous possible scenarios of divergence, we considered that these three scenarios represent the close relationships among groups and the most likely demographic scenarios during Pleistocene climate cycles (see Results).

We generated one million simulated datasets per scenario considering a generalized stepwise‐mutation model, a uniform prior distribution with 10–100,000 values for effective population sizes, and 100–50,000 generations for splitting events at t1, t2, and t3, depending on the population, and compared scenarios using DIYABC. The posterior probability of scenarios was assessed using a logistic regression on the 1% of simulated datasets closest to the observed data (Fontaine et al. 2013). For the best‐supported scenario, we performed a model checking procedure by applying a principal component analysis (PCA) on test statistic vectors to visualize the fit between simulated and observed datasets. The number of alleles, mean genic diversity, mean size variance, number of haplotypes, number of segregating sites and mean pairwise differences were used as summary statistics for each of the four groups, whereas for each group pair we used combined mean genic diversity, *F*_ST_, combined number of segregating sites and *N*_ST_. To assess confidence in scenario choice, we simulated 500 pseudo‐observed datasets (PODs) under each scenario to estimate Type I and Type II error rates (Robert et al. 2011). Finally, for the best‐supported scenario, point estimates for demographic and temporal parameters were obtained by local linear regression on the 1% of simulations closest to the observed dataset (Cornuet et al. 2008, 2014).

### Palaeodistribution modelling

We constructed species distribution models (SDM; Elith et al. 2011) to explore the potential distribution of *L. amethystinus* under current climatic conditions and to predict where the suitable conditions resided during the LGM (21,000–18,000 years ago) and LIG (120,000–140,000 years ago) and whether the conditions for range expansion and population connectivity occurred. We assembled a dataset of occurrences for *L. amethystinus* from georeferenced museum specimens obtained through http://vertnet.org and the Global Biodiversity Information Facility (GBIF, http://data.gbif.org/species/browse/taxon), supplemented with records from field collection. After careful verification of every data location and removing duplicate occurrence records, we restricted the dataset to unique records for the analyses, leaving 109 unique presence records for *L. amethystinus*. These localities sample the entire distribution range of each species. Distribution records were input into and analysed with the maximum entropy algorithm in MAXENT ver. 3.2.2 (Phillips et al. 2006) using the *dismo* ver. 1.0‐5 package (Hijmans et al. 2014) in R ver. 3.0.3 (R Development Core Team; http://www.r-project.org/) to infer the SDMs. Present‐day temperature and precipitation data (BIO1–BIO19 variables) were drawn as climate layers from the WorldClim database (ca. 1 km^2^; Hijmans et al. 2005). A principal components analysis (PCA) was carried out using SPSS ver. 17 for Mac (SPSS, Armonk, NY) to reduce the number of climatic variables and to minimize collinearity. We then ran a correlation analysis to eliminate correlated environmental variables using the program PAST ver. 2.12 (Hammer et al. 2001). When the correlation coefficient was higher than 0.8 the variables were considered highly correlated, and for each pair of correlated variables we selected the ones with the highest loadings on the first PC components. After removing the highly correlated variables, the remaining were used to generate the SDM model under current climate conditions using MAXENT (BIO4 =  Temperature Seasonality, BIO7 =  Temperature annual range, BIO12 =  Annual Precipitation, BIO17 =  Precipitation of Driest Quarter, BIO18 =  Precipitation of Warmest Quarter) with the default parameters for convergence threshold (10^−5^) and 500 iterations, ensuring only one locality per grid cell. We evaluated model performance using cross‐validation running a random dataset using 70% of the occurrence points for training and 30% to test the model and then estimating the area under the receiver operating curve (AUC) of the threshold‐independent receiving operating characteristic curve (ROC; Mertz 1978).

Resulting species distribution under current climate conditions was projected onto past climate scenarios, at the LGM (at ca. 2.5 arc‐min) and LIG (at 30 arc‐sec), using the *dismo* package (Hijmans et al. 2014) in R. Past climate layers were drawn from WorldClim webpage for two LGM scenarios (Braconnot et al. 2007): the Community Climate System Model (CCSM; Collins et al. 2004) and the Model for Interdisciplinary Research on Climate (MIROC; Hasumi and Emori 2004), and for the LIG (Otto‐Bliesner et al. 2006). The CCSM and MIROC climate models simulate different climate conditions, with cooler sea‐surface temperature conditions assumed in CCSM than in MIROC, resulting in higher annual precipitation in CCSM than in MIROC (Otto‐Bliesner et al. 2007; Ramírez‐Barahona and Eguiarte 2014).

### Morphological variation

Three body measurements were obtained from each of 242 hummingbirds (131 males and 111 females) from museum specimens (Acknowledgments) using a dial calliper with a precision of 0.1 mm: exposed culmen (from the base of the bill to the tip of the upper mandible); wing chord (the distance from the carpal joint to the tip of the longest unflattened primary); and tail length (from the uropygial gland to the tip of the longest rectrix). All measurements were taken by CG. Morphological data were tested for normality and log‐transformed (*x* + 1) before statistical analysis.

We performed a multivariate analysis of variance (MANOVA) followed by one‐way ANOVAs to examine morphological variation among groups of populations with SPSS ver. 17.0 for Mac (SPSS).

### Gorget color variation

Reflectance measurements were taken from one feather of the gorget (the area of bright iridescent coloration in between the chest and throat) obtained from 85 male museum specimens to which we had permission for plucking (see Acknowledgments). Bright iridescent coloration of feathers in gorgets, produced by nanoscale arrangements of multilayer stacks of keratin, melanosomes and air within feather barbules (Greenewalt et al. 1960; Maia et al. 2013a; Eliason et al. 2015), are lacking entirely in *L. amethystinus* females. Feather coloration between the chest and throat of females is dusky cinnamon, a pigment‐based color that is likely constrained in their expression by metabolic pathways (Prum et al. 2012; Maia et al. 2013a) and, therefore, their color variation was out of the scope of this study. We only measured iridescent gorget feathers based on apparent use of these ornaments in male courtships and agonistic displays and the nonornamental iridescent green back feathers is perhaps not sexually selected but under natural selection to aid in crypsis while perched (Ornelas et al. 2002; Meadows et al. 2012). Because measured feathers were iridescent (reflectance spectra change depending on the geometry of illumination and perception), reflectance measurements were taken at a 45° angle, while the illumination angle was fixed at 90° (Osorio and Ham 2002; Parra 2010). We performed preliminary measurements on possible angles to ensure that measurements taken at 45° captured the maximum percentage of reflectance. We used a RPH reflection probe holder (Ocean Optics, Dunedin, FL) that contained two apertures, at 45° and 90°, in which the light reception and light emission fibre cables were placed respectively. The probe holder was perfectly fitted in a box covered by black foamy material. The individual feather was placed in the box, flattened with a transparency sheet with a 5 mm diameter hole to allow the light sources reach the measured area directly (Parra 2010). Reflectance measurements were taken with an Ocean Optics Jaz‐El200 spectrophotometer (Ocean Optics) coupled with a Premium QR600‐7‐SR125F optic fibre and a Miniature pulsed xenon light source for UV‐VIS (220–750 nm) PX2 (Ocean Optics) as the illuminating light source. Measurements were taken relative to a WS‐1 diffuse white standard (Ocean Optics).

The raw data files of measured reflectance spectra of 85 males were first loaded and organized for further analysis in the R package PAVO ver. 0.5–1 (Maia et al. 2013b). Then the electrical noise arising from the spectrometer was removed using local regression smoothing implemented by the loess.smooth function in R, wrapped in the opt = “smooth” argument of procspec (span* *=* *0.25). We plotted the resulting spectral data to visualize the mean reflectance curves for gorgets from each genetic group (or subspecies). Because we detected two peaks from the spectral curve (see Results), we extracted a metric of hue (wavelength of peak reflectance) independently from the UV/blue peak (between 300 and 500 nm) and from a second peak situated beyond 600 nm. To test for differences among groups, we conducted one‐way ANOVAs with the hue data for each of the peaks and then plotted to visualize differences among genetic groups in R ver. 1.22 (http://www.r-project.org/). Although hues of the second peak are likely outside the range of avian vision, we provide these data to show that there is a previously unnoticed strong signal probably perceived by hummingbirds.

To explore how the birds perceive these colors, we also analysed the spectral data under the avian tetrahedral color space accounting for attributes of the color vision of the signal receiver. The avian visual system is comprised of four cone types, and under the color space model, all colors can be located in the volume of a tetrahedron, in which each of the four vertices represents the maximum stimulation of that particular cone type (Maia et al. 2012, 2013b). Using the R package PAVO, we first generated a three‐dimensional plot indicating the location of each point in the color tetrahedron, and then calculated the Cartesian coordinates (*X*,* Y*,* Z*) for the points in the tetrahedral color space, the angles theta and phi (h.theta, h.phi) in radians, which determine the hue of the color, the r vector (r.vec), which measures saturation or the distance from the achromatic centre, the maximum r vector (r.max) achievable for the color's hue, and the r.achieved, which measures the relative r distance from the achromatic centre in relation to the maximum distance achievable (r.vec/r.max). These are receiver‐centric variables that represent reflectance spectra in the avian tetrahedral color space (Maia et al. 2012). To test for differences between groups, we conducted a MANOVA with these coordinates and colorimetric variables followed by one‐way ANOVAs in R. Finally, we calculated the volumes occupied by each group’ gorget plumage, as well as their overlap using the R package PAVO.

## Results

### Genetic diversity and phylogeographical structure

The alignment of concatenated *ND2* and cyt *b* genes yielded a total of 751 bp with 96 variable sites (*S*) (Table 1). Varying levels of genetic diversity were observed among groups of populations, with the lowest number of haplotypes being 2 (TUX) with four samples and the highest number being 26 (SMO) with 70 samples (Table 1). Haplotype diversity (*h*) and nucleotide diversity (π) values were high, with samples from CHIS containing the highest levels (0.98, 0.0073) followed by those from SMO (0.88, 0.0037) respectively (Table 1).

**Table 1:** Summary statistics of *Lampornis amethystinus* populations grouped based on mountain geography. *N *= number of sequences, *N*_*H*_ **=* *number of haplotypes, *h *= gene diversity, *π *=* *nucleotide diversity, *D*_T_ **=* *Tajima's *D*,* F*_*S*_ **=* *Fu's *F*s, SDD* *=* *differences in the sum of squares or mismatch distribution, Hri* *=* *Harpending's raggedness index. Positive values for *D*_T_ and *F*_*S*_ are indicative of mutation‐drift‐equilibrium, which is typical of stable populations, whereas negative values that result from an excess of rare haplotypes, indicate that populations have undergone recent expansions, often preceded by a bottleneck. Significantly negative values (at the *P *=* *0.05 level for Tajima's *D* test and *P *<* *0.02 for *Fs* test; Excoffier and Lischer 2010) in both tests reveal historic demographic expansion events. Significant (*P *≤* *0.05) SSD and Hri values indicate deviations from the sudden expansion model. Values that are consistent with demographic expansion are shown in bold

Cloud forest area	*N*	*N*_*H*_	*h*	*π*	*D*_T_	*F*s	SSD	Hri
SMO	70	26	0.88 ± 0.02	0.0037 ± 0.002	**−1.566** *	**−5.789** **	**0.0169** *	**0.1564** *
TUX	4	2	0.50 ± 0.26	0.0033 ± 0.002	n.a.	n.a.	n.a.	n.a.
TMVB	19	6	0.74 ± 0.06	0.0023 ± 0.001	**−1.718** *	**−**1.084	0.0081	0.4931
SMS	37	17	0.88 ± 0.03	0.0029 ± 0.001	**−1.517** *	**−3.238** **	0.2219	0.2065
CHIS	27	20	0.98 ± 0.01	0.0073 ± 0.003	**−1.512** *	**−8.436** ***	0.0036	0.0531

n.a., not available.

Region abbreviations are as follows: SMO, Sierra Madre Oriental; TUX, Sierra de Los Tuxtlas and Sierra de Santa Marta; SMS, Sierra Madre del Sur (Sierra de Miahuatlán, Oaxaca and Guerrero); TMVB, Trans‐Mexican Volcanic Belt; CHIS, Chiapan Highlands separated by the Central Depression that together with Guatemala and El Salvador form the region TIH (Trans‐Isthmian Highlands).

*P <* *0.05, **P <* *0.01, ***P <* *0.001.

**Figure 2:**
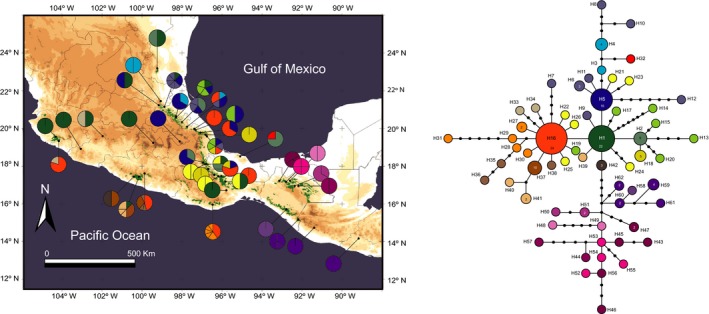
Geographic distribution and statistical parsimony network of the *ND2* and cytochrome *b* haplotypes of 157 *Lampornis amethystinus*. Current natural range of cloud forests (indicated by green shading) is overlaid on a relief map of Mexico. The current species’ range is restricted to isolated populations parallel to that of the cloud forest range shown on the map. Pie charts represent haplotypes found in each sampling locality. The size of sections of the pie charts corresponds to the number of individuals with that haplotype. Population numbers are the same as those used in Tables S1 and S2.

Sixty‐two mtDNA haplotypes were recovered, with most localities exhibiting more than one haplotype (Fig. 2; see also Table S2). Statistical parsimony retrieved a single network with two main mtDNA haplogroups separated by the IT without haplotype sharing and connected by five mutations (Fig. 2). Most populations west of the IT had individuals with haplotypes H16, H1, or H5, the three most common haplotypes in the study (Fig. 2). The rest of the haplotypes were exclusively found in individuals from populations within each of the geographic regions (e.g., H32 Sierra de Los Tuxtlas; H27–H31 Sierra de Miahuatlán; H35–H41 Guerrero; Fig. 2; Table S2). East of the IT none of the haplotypes were dominant, and the different haplotypes were characteristic of each region (H43–H57 cCHIS; H58–H62 pCHIS; Fig. 2; Table S2).

The AMOVA results for mtDNA showed that 61.4% of the genetic variation was explained by differences within populations and 38.6% by differences between populations when all locations were treated as a single group (Table 2). Genetic differentiation and population structure was highest and significant (*F*_CT_ **=* *0.53) when populations are grouped as separated by the IT (Table 2). When sampling sites were grouped by mountain geography, a significant but smaller proportion of the variation (34%) was attributed to differences between groups (Table 2). Pairwise comparisons of *F*_ST_ values were all significant when groups of populations are compared, with the exception of the TUX‐SMO, TUX‐TMVB, and TUX‐SMS comparisons after Bonferroni correction. The strongest relationships were observed between CHIS and the rest of the groups of populations (Table S3).

**Table 2:** Results of the AMOVA model on *Lampornis amethystinus* populations with no groups defined a priori (A), and grouped into groups of populations separated by the Isthmus of Tehuantepec (B) or into five groups based on mountain geography (C)

	d.f.	Sum of squares	Variance components	Percentage of variation	Fixation indices
(A) No groups defined					
Among populations	35	109.82	0.53	38.61	
Within populations	121	102.12	0.48	61.39	*F*_ST_ * *=* *0.38***
Total	156	211.93	1.37		
(B) Isthmus of Tehuantepec					
Among groups	1	54.22	1.17	53.34	*F*_CT_ * *=* *0.53***
Among populations within groups	34	55.59	0.18	8.33	*F*_SC_ * *=* *0.18***
Within populations	121	102.12	0.84	38.32	*F*_ST_ * *=* *0.62***
Total	156	211.93	2.20		
(C) Geographic region					
Among groups	4	64.05	0.52	34.43	*F*_CT_ * *=* *0.34***
Among populations within groups	31	45.76	0.15	9.94	*F*_SC_ * *=* *0.15***
Within populations	121	102.12	0.84	55.63	*F*_ST_ * *=* *0.44***
Total	156	219.93	1.52		

***P <* *0.0001.

Across groups of populations (TUX group excluded due to small sample size), the mean number of alleles per locus and allele richness values were moderately high and similar among groups of populations, ranging from 7.4 (SMS) to 5.4 (TMVB) and from 5.4 (CHIS to 4.6 (SMO) respectively (Table S4). Observed heterozygosity values ranging from 0.56 (TMVB) to 0.47 (CHIS) did not consistently deviate from H–W equilibrium (Table S4). The mean expected heterozygosity ranged from 0.76 in the TMVB group to 0.59 in the CHIS group (Table S4). According to MICROCHECKER, two localities departed from H–W equilibrium after Bonferroni corrections at locus CACU16‐1 and HumB15, probably due to the presence of null alleles. No significant linkage disequilibrium was detected in any of the population‐loci comparisons after Bonferroni corrections (Table S5).

**Figure 3:**
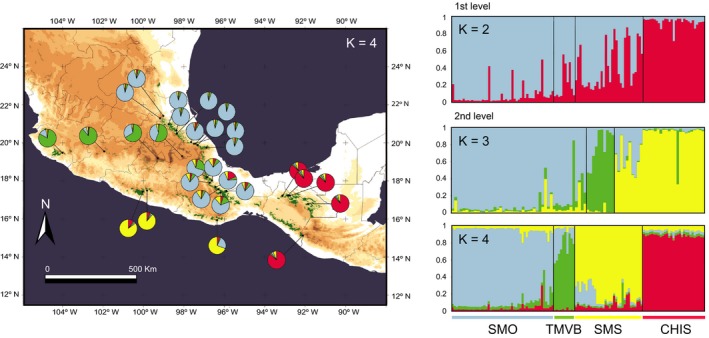
Assignment probabilities of *Lampornis amethystinus* individuals to putative population clusters at *K *=* *2 using STRUCTURE (1st level), and assignment probabilities of individuals to putative population clusters at *K *=* *3 and *K *=* *4 (2nd level) using STRUCTURE with individuals east of the IT excluded (CHIS populations). Each individual is represented by a vertical line that is partitioned into *K* colored sections, with the length of each section proportional to the estimated membership coefficient. TUX population was not included due to the small sample size.

Significant microsatellite‐based genetic subdivision was detected among sampling localities (global *F*_ST_ estimate* *=* *0.0635, *P *<* *0.05). Pairwise *F*_ST_ values from comparisons between groups of populations were all statistically significant, with the weakest relationship observed between SMO and SMS (Table S3). The clustering using STRUCTURE not only revealed substantial phylogeographic structure but also recovered hierarchical relationships among groups of populations. Two peaks of Δ*K* were detected, and the break in the slope of the distribution of *L*(*K*) was at *K *=* *2 and *K *=* *4. When *K *=* *2, individuals from the CHIS group were firstly separated from others west of IT (Fig. 3), so concordant with mtDNA sequence analyses. Some level of uncertainty in assignment, likely reflecting allele sharing due to incomplete lineage sorting and/or admixture, was observed. For *K *=* *2, a moderate number of individuals (ca. 9) from the SMS geographic group have large assignment *Q* values (>0.6) indicating their membership in what is otherwise considered the CHIS gene pool (Fig. 3). When *K *=* *4, further substructuring was observed generally concordant with the SMO, TMVB, SMS, and CHIS geographic groups, with high individual assignment *Q* values observed in most cases averaging assignment values of 0.86, 0.74, 0.81, and 0.88 to genetic clusters SMO, TMVB, SMS, and CHIS respectively. To corroborate the structure detected with this first analysis, a subsequent analysis was conducted excluding individuals from the CHIS group from the dataset to determine if any additional substructure was detected. This second analysis was performed with the same settings used in the first analysis, and a single peak was detected by Δ*K* at *K *=* *3. The three clusters detected corresponded to SMO, TMVB, and SMS geographic groups, which confirmed the genetic substructure detected by the first analysis (Fig. 3).

### Demographic history

Most of the neutrality test values were negative and significant (except Fu's *Fs* for TMVB), indicating historic demographic expansion events (Table 1). In the mismatch distribution, the hypothesis of sudden demographic expansion (SSD and Hri values) was rejected, indicating that populations have not experienced recent rapid population expansion except for the SMO (Table 1).

The BSP confidence intervals indicated that groups of populations experienced no significant population size changes across time, except a subtle increase in population size before the LGM in the SMO group (Fig. S2).

### Divergence time estimation

The BEAST analysis grouped all *L. amethystinus* samples in a well‐supported monophyletic group (PP* *=* *1.0; Fig. S3). Within this clade, the phylogenetic relationships among haplotypes were split between populations separated by the IT. Individuals from populations east of the Isthmus form two highly supported groups (PP* *=* *0.99), one corresponding to the *salvini* subspecies south of the Motagua‐Polochic‐Jocotán fault system (MPJ) and the other with samples north of the MPJ fault system in Chiapas. Relationships between samples from the Sierra Madre del Sur (*margaritae* and *circumventus*) and those from the Sierra Madre Oriental, Sierra de Los Tuxtlas and the Trans‐Mexican Volcanic Belt (*amethystinus* and *brevirostris*) were poorly resolved. Diversification of mtDNA haplotypes in *L. amethystinus* began in the Pleistocene with the split between IT lineages estimated at 1.49 MYA (95% highest posterior density, HPD: 2.39–0.57 MYA) and Late Pleistocene ages for the split between CHIS populations separated by the MPJ fault system (0.57 MYA, 95% HPD 1.01–0.17 MYA; see phylogram in Fig. S3).

### Historical and contemporary gene flow

IMa results are summarized in Table 3 and reported as the average of two runs of mean parameter estimates and the 90% highest posterior densities (HPD) intervals of each parameter. For the comparison between groups of populations separated by the IT, the western population size is estimated to be larger than the eastern population but smaller than the population size of the ancestral population (Table 3). The same pattern was observed for comparisons between amethyst versus blue and SMO versus TMVB populations (Table 3). Divergence time between populations separated by the isthmus was estimated at ca. 11 ka BP (95% HPD 24.5–4.4 ka BP). Time since divergence between amethyst versus blue and SMO versus TMVB was estimated at 3.2 (95% HPD 23.1–1.6 ka BP) and 2.8 ka BP (95% HPD 23.8–1.1 ka BP) respectively (Table 3). When testing for migration following the split between populations, migration in both directions of the Isthmus is low (Table 3) and migration between amethyst and blue and between SMO and TMVB was higher and asymmetrical; migration following the split was higher from blue to amethyst and from TMVB to SMO than in the opposite directions (Table 3).

**Table 3:** Results of isolation‐with‐migration model (IMa) for the splits between groups of populations of *Lampornis amethystinus*

	Model parameter estimates					
	*q*_1_	*q*_2_	*q*_*a*_	*t*	*M*_2‐to‐1_	*M*_1‐to‐2_
West vs. east of IT						
Mean	6.2304	3.9441	22.8639	4.415	0.609	0.799
HPD95Lo	4.5156	2.7525	3.8106	1.76	0.102	0.034
HPD95Hi	8.6502	6.1143	59.1413	9.8	0.78	0.786
Amethyst vs. blue						
Mean	4.5876	3.0133	23.2372	1.275	4.396	0.502
HPD95Lo	2.9193	1.7999	9.2220	0.645	0.6	0.067
HPD95Hi	7.5613	5.1773	114.8512	9.255	7.548	4.34
SMO vs. TMVB						
Mean	6.4739	0.5901	16.1594	1.125	4.997	1.83
HPD95Lo	4.0813	0.3177	4.4484	0.47	1.327	0.325
HPD95Hi	17.4866	1.9972	86.3801	9.51	4.95	9.555

	Demographic parameter estimates					
	$N_{e_{1}}$	$N_{e_{2}}$	$N_{e_{a}}$	*t*	*M*_2‐to‐1_	*M*_1‐to‐2_
West vs. east of IT						
Mean	1419.052	898.318	5207.541	11,061	1.896	1.577
HPD95Lo	1028.485	627.543	867.923	4409	0.229	0.046
HPD95Hi	1970.180	1392.620	13470.186	24,552	3.391	2.402
Amethyst vs. blue						
Mean	1044.883	686.3283	5292.5643	3194	10.083	0.757
HPD95Lo	664.9186	409.9612	2100.4378	1616	0.875	0.061
HPD95Hi	1722.1925	1179.1951	26158.8156	23,187	28.536	11.235
SMO vs. TMVB						
Mean	1474.512	134.403	3680.506	2818	16.176	0.539
HPD95Lo	929.579	72.362	1013.179	1177	2.709	0.052
HPD95Hi	3982.793	454.887	19674.165	23,826	43.279	9.542

Model parameters indicate estimates without use of molecular rate of evolution for six parameters (IMa output values). Demographic rates represent parameters scaled to rates of molecular evolution. Values are averages of two runs of mean parameter estimates and the 95% highest posterior densities (HPD) intervals of each parameter: effective population sizes (*N*_*e*_, individuals), migration rates (*N*_*m*_, migrants per generation), estimated time since divergence (*t*, years). Population size (*N*_*e*_) based on the average generation time (*T*) of 3.08 years for a high (0.52) annual adult survival rate.

### Analyses of population history with coalescence models

**Figure 4:**
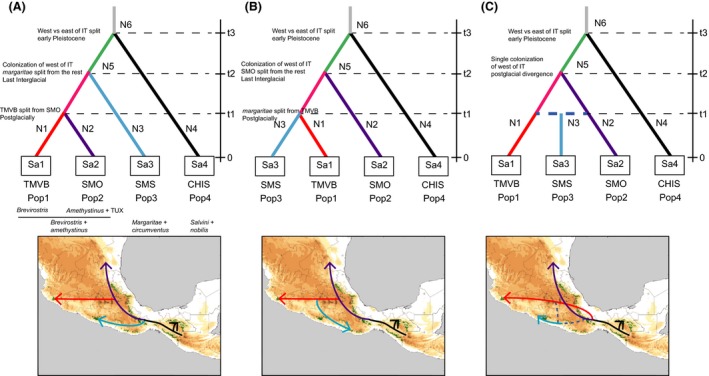
Competing demographic scenarios of *Lampornis amethystinus* divergence and admixture. (A) *Isolation split model 1* (Sc1) predicts that TMVB (Pop1) merged with SMO (Pop2) at t1 then SMO merged with SMS (Pop3, *margaritae*) at t2 and subsequently with CHIS east of IT (Pop4) at t3. This scenario was expected to be the most likely according with hierarchical STRUCTURE and BEAST analyses. (B) *Isolation split model 2* (Sc2) is similar to the previous one but predicts that SMS (Pop3) merged with TMVB (Pop1) at t1 then TMVB merged with SMO (Pop2) at t2 and subsequently with CHIS east of IT (Pop4) at t3. (C) *Isolation with admixture model* (Sc3) consisted of the same basal split between CHIS (Pop4) and the rest of groups west of IT described in previous scenarios but includes a hybridization/lineage fusion event in which SMS (Pop3) is the descendent of admixture between TMVB (Pop1) and SMO (Pop2) at t1, then Pop1 merged with Pop2 at t2, and subsequently with Pop4 at t3. t# refers to timescale expressed as generation time and N# to effective size of the corresponding population (N_CHIS_, N_SMS_, N_SMO_, N_TMVB_, or the ancestral populations) during each time period (e.g., 0–t1, t1–t2, t2–t3).

In DIYABC analysis for the combined mtDNA and microsatellite dataset, scenario 1 was found to be the most likely (Fig. 4, Fig. S4), with the highest posterior probability for this model (0.5657) and 95% confidence intervals (95% CI: 0.5538–0.5775) without overlap with those obtained for scenarios 2 (0.1233, 95% CI: 0.1087–0.1380) and 3 (0.3110, 95% CI: 0.3015–0.3204). This is corroborated by model checking using the PCA, which yielded a large cloud of data from the prior and observed datasets centred on a small cluster from the posterior predictive distribution, suggesting that the best supported scenario explained the observed data well (Fig. S4). Analyses to estimate confidence in scenario choice based on 500 PODs indicate that Type I and Type II errors (0.29 and 0.21, respectively) for the best‐supported scenario were relatively low. Under scenario 1, posterior mean parameter estimates indicated that divergence between TMVB and SMO occurred 1320 years BP (t1), the split between SMS and SMO‐TMVB (t2) around 3300 years BP, while the CHIS and SMS‐SMO‐TMVB lineage split around the end of the LGM (t3), 16,290 years BP (Table S6), assuming a 3‐year generation time of *L. amethystinus*. In accordance with the isolation and migration model, ABC estimated a smaller *N*_*e*_ for the descendant populations compared with both N5 and N6 ancestral populations, and a smaller *N*_*e*_ for the TMVB compared with the CHIS, SMS, and SMO populations (Table S6). Estimated mean mutation rates of mtDNA and microsatellites were estimated to be 0.000187 and 0.00620 respectively (Table S6). Most of the summary statistics including the number of alleles, number of haplotypes, mean size variance and *F*_ST_ or *N*_ST_ values for any population‐pair estimated using the acquired posterior distributions were not statistically different from the observed values (Table S7), suggesting that the scenario‐prior combination of scenario 1 is a good fit. However, mean genic diversity, combined mean genic diversity, number of segregating sites, and mean pairwise differences were underestimated and significantly differed from observed values (Table S7).

### Palaeodistribution modeling

The current distribution model of *L. amethystinus* was supported by high predictive power (AUC* *=* *0.979; Fig. S5). The LIG and LGM (CCSM and MIROC) distribution models yielded similar inferences and showed past conditions of suitable habitat somewhat similar to current distribution restricted to mountain ranges (Fig. S5). Overall, estimated potential distribution of *L. amethystinus* indicates that this species had a relatively stable distribution range during the last 140,000 years BP (Fig. S5).

### Morphological variation

For males, the MANOVA of morphological variables followed by univariate ANOVAs showed significant differences among populations grouped by geographic region (Wilks’ Lambda* *=* *0.526, *F*_12, 328_ **=* *7.52, *P *<* *0.0001; exposed culmen, *F*_4, 130_ **=* *4.38, *P *=* *0.002; wing chord, *F*_4, 130_ **=* *13.62, *P *<* *0.0001; tail length, *F*_4, 130_ **=* *12.34, *P *<* *0.0001), in which SMS and TMVB males were larger with shorter bills than those across SMO, TUX, and CHIS (Fig. S6). For females, the same pattern of morphological differentiation was observed (Wilks’ Lambda* *=* *0.542, *F*_12, 275_ **=* *5.98, *P *<* *0.0001; exposed culmen, *F*_4, 106_ **= 2.86, *P *=* *0.027; wing chord, *F*_4, 130_ **=* *11.66, *P *<* *0.0001; tail length, *F*_4, 130_ **=* *10.48, *P *<* *0.0001), with larger females with shorter bills from SMS and TMVB than those across SMO, TUX, and CHIS (Fig. S6).

### Gorget color variation

**Figure 5:**
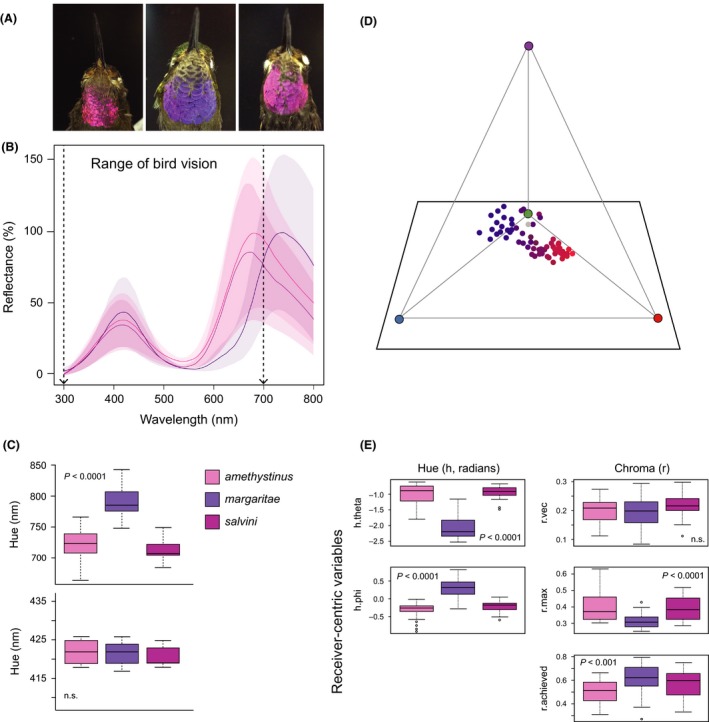
Gorget color variation of *Lampornis amethystinus* iridescent feathers. (A) *Lampornis amethystinus amethystinus* (left), *L. amethystinus margaritae* (centre), *L. amethystinus salvini* (right). (B) Mean smoothed spectra curves for gorget iridescent feathers, with corresponding standard deviations shown as shaded areas. (C) Box plots showing subspecies differences in mean hue (wavelength of maximum reflectance) of gorget feathers between 550 and 1000 nm (above) and between 300 and 500 nm (below). (D) Tetrahedral color space plot of variables using a sensory‐based analysis indicating the location of each point in a three‐dimensional space, with blue points corresponding to *margaritae* individuals and the remaining purple‐to‐red points to *amethystinus* and *salvini* indistinctly. (E) Receiver‐centric variables (hue and chroma) of a tetrahedral color space analysis (see Methods for detailed description of variables).

Reflectance spectra of the gorget feathers of *L. amethystinus* showed two reflectance peaks: a main peak situated around 700 nm and a smaller peak situated around 400 nm (Fig. 5A,B). The combination of both peaks of maximum reflectance (hue) results in the colors we perceive, and in theory the birds. The gorgets of *amethystinus* and *salvini* looks amethyst‐pink because of the combination of blue and in greater proportion red colors, however, the gorgets of *margaritae* looks blue‐purple because the red peak is almost completely outside the range of human vision and therefore predominates the blue. Significant differences were noted in the wavelength of hue between subspecies in the red peak, but not in the UV/blue peak (one‐way ANOVAs; red peak, *F*_2, 82_ **=* *99.94, *P *<* *0.001; UV/blue peak, *F*_2, 82_ **=* *1.36, *P *>* *0.05; Fig. 5C). Mean hues of the *margaritae* group in the red peak were around 780 nm, whereas those for *amethystinus* and *salvini* were at 715 nm approximately. On the other hand, the UV/blue peak in the three groups was around 420 nm.

The tetrahedral representation of bird vision indicating the location of each point in a three‐dimensional space is shown in Fig. 5D, with blue points corresponding to *margaritae* individuals and the remaining purple‐to‐red points to *amethystinus* and *salvini* indistinctly. We found significant differences in receiver‐centric variables (hue and chroma) between blue‐ and amethyst‐throated groups of populations (MANOVA; Wilks’ Lambda* *=* *0.1008, *F*_16, 150_ **=* *20.15, *P *<* *0.0001), and univariate ANOVAs yielded significant group differences for all coordinates in the tetrahedron, hue and chroma variables, except r.vec (*X*,* F*_2, 82_ **=* *87.96, *P *<* *0.0001; *Y*,* F*_2, 82_ **=* *4.21, *P *<* *0.01; *Z*,* F*_2, 82_ **=* *97, *P *<* *0.0001; h.theta, *F*_2, 82_ **=* *109.6, *P *<* *0.0001; h.phi, *F*_2, 82_ **=* *62.97, *P *<* *0.0001; r.vec, *F*_2, 82_ **=* *1.71, *P *>* *0.05, r.max, *F*_2, 82_ **=* *11.42, *P *<* *0.0001, r.achieved, *F*_2, 82_ **=* *7.43, *P *<* *0.001, Fig. 5E). In all significant comparisons *margaritae* was different from *amethystinus* and *salvini*.

Finally, no overlap in the tetrahedral color volume between *margaritae* and *amethystinus*, and between *margaritae* and *salvini* were observed. On the contrary, 0.73% of the tetrahedral color volume overlaps by *amethystinus* and *salvini*. This suggests that *margaritae* versus *amethystinus* and *salvini* occupy different “sensory niches” (Maia et al. 2012), whereas *amethystinus* and *salvini* overlap.

## Discussion

### Population genetic structure

Our study revealed that *L. amethystinus* exhibits high geographical genetic structure and low levels of population connectivity in Mesoamerica. We found high differentiation in mitochondrial DNA and microsatellite data, with most groups of populations showing significant pairwise differences among them. For microsatellite data, SMO, TMVB, SMS, and CHIS populations corresponding to mountain ranges are significantly differentiated in multiple comparisons, suggesting geographic isolation. Clustering analyses support a strong population differentiation, with both nuclear and mitochondrial markers indicating a clear east‐west division along the IT. Additionally, microsatellite data suggests further subdivision structured along the main mountain ranges in the region, with four populations being the best fit for the data. According to the ABC analysis, the most strongly supported scenario was lineage divergence after the LGM (scenario 1) when tested against competing scenarios and based on combined mtDNA and microsatellite data. Under this scenario of recent divergence, DIYABC analysis provided good support for an older divergence between groups separated by the IT (CHIS group and the rest of groups) around the end of the LGM (16,290 years ago; Table S6), and later divergence between SMS and SMO+TMVB around 3300 years ago, and subsequently between SMO and TMVB groups around 1320 years ago. The DIYABC divergence time estimates supporting postglacial divergence of *L. amethystinus* groups are very similar to estimates for the joint dataset of mtDNA and microsatellites using IMa, in which convergence was confidently achieved. The reconstruction of genetic histories for recently diverged lineages is hindered by stochasticity in the coalescent process and the confounding influences of migration and incomplete lineage sorting (Arbogast et al. 2002). However, the thorough sampling from throughout the species range (43 localities, 4–3 individuals per locality on average for mtDNA sequences and microsatellites, respectively) and a large sample size per population (10 localities per population on average, 38–30 individuals per population for mtDNA sequences and microsatellites, respectively) overcome these difficulties (e.g., Funk and Omland 2003; McCormack et al. 2008).

High levels of haplotype, and nucleotide and allelic diversities throughout most of the populations of *L. amethystinus* suggest large population sizes and few founder effects or population bottlenecks. The exception to this is the TUX population, which exhibits reduced haplotype and nucleotide diversity for mitochondrial markers relative to the other groups of populations likely due to the small sample size. Patterns of high population structure are expected to this cloud forest‐adapted hummingbird species. Other hummingbird species in Mesoamerica exhibit limited population structure despite the presence of proposed barriers to dispersal. Studies of *Campylopterus curvipennis* (González et al. 2011) and *Amazilia cyanocephala* (Rodríguez‐Gómez et al. 2013), that are partially codistributed in the cloud forests with *L. amethystinus* but have wider altitudinal ranges, found similarly high levels of genetic diversity but population differentiation was only significant between groups of populations separated by the IT. Limited differentiation has also been found in other hummingbird species residents of the Mesoamerican lowlands (*Amazilia tzacatl*; Miller et al. 2011; *Phaethornis longirostris*; Arbeláez‐Cortés and Navarro‐Sigüenza 2013) or montane hummingbird species with seasonal migration (*Selasphorus platycercus*; Malpica and Ornelas 2014). However, in the Andean cloud forests *Adelomyia melanogenys* exhibits significant differentiation between coastal populations and populations on either side of the Andes (Chaves et al. 2007). Overall, *L. amethystinus* population connectivity seems to be primarily limited by interglacial isolation of mountain ranges in Mesoamerica, which is reasonable for a resident hummingbird species restricted to the upper cloud forests in the region.

### Glacial/interglacial cycles and in situ divergence

During the LGM, cloud forest‐adapted species expanded their distribution to the lowlands and their distribution contracted allopatrically during the interglacials according to LGM simulations and the available paleodata for Mesoamerica (Graham 1999; Still et al. 1999). The demographic and distributional changes predicted by the expanding–contracting cloud forest archipelago model during the LGM depend on the regional precipitation regimes (Ramírez‐Barahona and Eguiarte 2013). Contrary with predictions of the moist forests model or the dry refugia model (Ramírez‐Barahona and Eguiarte 2013), our species distribution models predicted that *L. amethystinus* populations apparently remained in situ primarily within the current fragmented distribution of the cloud forest. Furthermore, divergence events for geographically persistent and structured populations with limited gene flow trace back to the LGM or earlier (interglacial divergence).

After the LGM, species that expanded and recolonized the species’ former fragmented range with the onset of warmer and more humid conditions are expected to exhibit three characteristics: (1) a star‐like haplotype network with many low frequency single haplotypes separated from high frequency central haplotypes by few mutational steps, (2) low levels of genetic differentiation between populations, and (3) a mismatch distribution of pairwise differences among haplotypes indicating a sudden increase in expansion from a single population. These signals of expansion from a single population have been reported in the partially codistributed *Amazilia cyanocephala* hummingbird species (Rodríguez‐Gómez et al. 2013) as well as codistributed cloud forest‐adapted plant species, including the hummingbird‐pollinated and bird‐dispersed *Palicourea padifolia* (Gutiérrez‐Rodríguez et al. 2011) and the epiphytic bird‐dispersed *Rhipsalis baccifera* (Ornelas and Rodríguez‐Gómez 2015). In contrast to the aforementioned studies, the results of the haplotype networks analysis, the AMOVA and pairwise comparisons of *F*_ST_ values based on both mtDNA and microsatellite data suggests the presence of a strong population genetic structure, indicating that the genetic differentiation of *L. amethystinus* in Mesoamerica resulted from the geographical isolation of montane cloud forest areas surrounded by drier lowland areas. In addition, this scenario is supported by the modeled paleodistribution that predicted that *L. amethystinus* populations apparently remained in situ during the LGM (see also Ornelas et al. 2015). This basin–mountain geographical pattern would have created a significant barrier to gene flow and the spread of the species, and the more arid conditions of the lowlands would have caused habitat fragmentation and a high degree of population isolation for *L. amethystinus* during the interglacial periods. The demographic and genetic expectations of the dry refugia model for cloud forest taxa include the following characteristics: (1) a star‐shaped haplotype genealogy, (2) demographic expansion and loss of genetic diversity, and (3) marked genetic structuring of populations (Ramírez‐Barahona and Eguiarte 2013). However, high levels of genetic variation in most of the geographical groups of *L. amethystinus* is not consistent with the dry refugia model in that postglacially colonized regions are expected to have reduced levels of genetic variation.

Private haplotypes mostly dominated the geographical groups, and the Fu's *Fs*, Tajima's *D*, mismatch distribution and BSP indicated no significant evidence of population expansions in mtDNA sequences of *L. amethystinus*. Supporting the dry forest refugia model, the few widespread haplotypes in the networks had frequencies that differed dramatically among the regions. Furthermore, the distribution of haplotype diversity and our geneflow estimates suggest that genetic divergence among groups of populations of *L. amethystinus* in the corresponding cloud forest areas is due to restricted gene flow across low elevation barriers. This pattern is more complex than that predicted by the dry refugia model, and has been reported in hummingbird‐pollinated *Moussonia deppeana* (Ornelas and González 2014) that *L. amethystinus* specializes upon. Ornelas and González (2014) found high genetic differentiation with genetic structuring of *M. deppeana* populations, in which populations also remained in situ, and that the divergence events for geographically persistent and structured populations with limited gene flow traced back to the LGM or earlier (Ornelas and González 2014). Because these species have a mutually dependent interaction we would expect a shared history that resulted in geographically concordant phylogroups with contemporary distributions in the cloud forests delimited by ancient sources of vicariance. If both species have responded in concert to historical environmental processes throughout Mesoamerica, in particular similar ages for spatially congruent population divergences, then there should be evidence of congruent biogeographical responses from two interacting but phylogenetically and ecologically distinct species. The observed similar phylogeographical patterns of the interacting *M. deppeana* and its pollinating *L. amethystinus* hummingbird further provides the potential of applying explicit phylogeographical testing using a comparative ABC approach to assess concordance between demographic patterns and interspecific phylogeographical congruence (Cornuet et al. 2014).

Similar mtDNA divergence patterns have been described for other cloud forest‐interior co‐distributed bird species, with marked genetic differentiation between populations accompanied with plumage and/or song divergence (*Buarremon* brush‐finches, Cadena et al. 2007; Navarro‐Sigüenza et al. 2008; *Aulacorhynchus prasinus* toucanets, Puebla‐Olivares et al. 2008; *Chlorospingus ophthalmicus* common bush‐tanagers, Bonaccorso et al. 2008; Sosa‐López et al. 2013; *Cyanolyca* jays, Bonaccorso 2009; *Catharus frantzii* ruddy‐capped nightingale‐thrushes Ortiz‐Ramírez et al. 2016). The apparent discordance between mitochondrial and microsatellite datasets (stronger mtDNA divergence signal at the IT compared to the geographic model suggested by AMOVA) is not uncommon in birds (e.g., Funk and Omland 2003; Rheindt and Edwards 2011; Toews and Brelsford 2012). Using the ABC analysis, we found that scenario 1 was the most supported scenario when tested against competing scenarios based on the joint mitochondrial and microsatellite dataset. The first split in this scenario suggests that *L. amethystinus* populations diverged from south to north in Mesoamerica ca. 16,000 years BP. The second indicates a split between blue (SMS) and amethyst (SMO and TMVB) populations after the LGM. The third and final split illustrates a more recent split between SMO and TMVB populations ca. 1300 years ago (Fig. 4, Fig. S4). Our results support the suggestion that mtDNA reflects the evolutionary history of *L. amethystinus* throughout the late Pleistocene, whereas more variable microsatellites useful for studying recent evolutionary events better reflect previously undescribed postglacial divergence and contemporary gene flow patterns among populations.

The ‘total evidence’ approach we implemented here gave us an overview on the phylogeography of *L. amethystinus* species complex, while the ABC analysis proved to be extremely valuable to circumvent limitations of descriptive and other model based methods. For instance, the IM model does not allow changes in population size through the history of a population, the absence of a migration parameter in the BEAST model for fitting population size change poses another model violation or the populations separately analysed to assess whether populations evolved under neutrality (Fu's *Fs* test and Tajima's *D* tests), deviations from sudden population expansion (SSD and Hri index) or changes in effective population size (BSP) do not satisfy the assumption of panmixia (Rogers et al. 1996; Städler et al. 2009; Chikhi et al. 2010; Peter et al. 2010; Ho and Shapiro 2011; Heller et al. 2013). Our balanced sampling strategy whereby samples are distributed on several populations provides the best scheme for inferring demographic change over a typical time scale, might overcome potential violations of this assumption (e.g., Funk and Omland 2003; McCormack et al. 2008; Chikhi et al. 2010; Ho and Shapiro 2011; Heller et al. 2013). Thus, in our study, these methods were useful for estimating confidence intervals of population parameters, narrowing down the possibilities of diversification scenarios. In this context, the ABC simulation‐based approach using both mtDNA and microsatellite loci with a higher evolutionary rate so that there is appreciable variation among sampled individuals is a powerful way to choose among different more complex divergence scenarios, overcoming limitations of methods with fixed models containing a particular number of parameters.

### Divergence between blue‐ and amethyst‐throated populations

We found genetic, morphological, and plumage color differences between allopatric blue‐ and amethyst‐throated populations of *L. amethystinus* isolated by geographic barriers, a pathway to speciation (Coyne and Orr 2004). We detected strong differences between blue‐ and amethyst populations using a sensory‐based analysis with tetrahedral color space variables. This is the most appropriate approximation to understand differences among populations because premating isolation would depend on color as seen by females. Interestingly, we detected a strong signal beyond the range of avian color vision (>700 nm), especially in the blue populations, that has not been reported. The degree to which hummingbirds detect red hues is not well‐known, but results from this study suggest that hummingbirds probably see further into the infrared in more nuanced and complex ways than previously noticed. The marked phenotypic differentiation between allopatric blue‐ and amethyst‐throated populations and their recent divergence is not consistent with the idea that time spent in isolation should be the primary factor predicting phenotypic differentiation of otherwise ecologically similar allopatric populations (Price 2010; Winger and Bates 2015) and therefore, the recent divergence time between blue‐ and amethyst‐throated populations does not correspond to the amount of time populations need to be in isolation for substantial plumage divergence, approximately two million years to evolve in allopatry (Winger and Bates 2015). Our estimates of divergence times suggest that diversification of mtDNA haplotypes in *L. amethystinus* began in the Pleistocene 2.39–0.57 MYA with the split between lineages separated by the IT (Fig. S3), but the estimated time since divergence between blue‐ and amethyst‐throated populations occurred after the LGM according to IMa and DIYABC analyses. In addition, we found no significant gorget color differentiation between individuals from populations separated for the longest time at the IT according to our divergence time estimates. It is possible that the small amounts of gene flow observed across the IT might erase incipient phenotypic differences, and this gene flow pushed our estimates of divergence times toward present (Rheindt and Edwards 2011; Winger and Bates 2015). Genetic differentiation between populations of *Amazilia cyanocephala* separated by the IT was also found in the presence of small amounts of gene flow (Rodríguez‐Gómez et al. 2013). This hummingbird species, which is partially co‐distributed in the cloud forests with *L. amethystinus* but has wider altitudinal ranges, is monomorphic, lack iridescent gorgets, and has not phenotypically diverged because homogenizing gene flow and habitat connectivity across the isthmus (Rodríguez‐Gómez et al. 2013). These observations suggest that the distribution at higher elevations and confounding influences of gene flow do not fully explain divergence of iridescent gorgets between blue‐ and amethyst‐throated hummingbirds. Although genetic drift is one possible explanation for the marked differentiation between blue‐ and amethyst‐throated hummingbirds (Parra 2010), one alternative is that certain color classes, namely, structurally iridescent colors, are more malleable than others like pigment‐based metabolically constrained colors, as previously suggested by Maia et al. (2013a). If so, weakly divergent selection pressures on hummingbird individuals with structurally iridescent colors likely associated with mate choice and competition would suffice to a relatively fast differentiation, even in the presence of gene flow (Parra 2010; González and Ornelas 2014), compared to those lacking ornamental iridescent coloration that would require longer periods of geographic isolation.

In contrast with male plumage differentiation, we found greater change in morphometric traits of male and female *L. amethystinus* hummingbirds, with statistical differences between birds from SMS and TMVB and those from SMO, TUX, and CHIS (Fig. S5). However, the observed differences between groups of populations in morphometric traits, in which hummingbirds from SMS and TMVB were larger with shorter bills than hummingbirds in localities across the SMO, TUX, and CHIS, do not correspond to the geographically isolated sister groups of populations (e.g., groups separated by the IT) or sister groups that differ in throat color (e.g., blue‐ vs. amethyst‐throated populations west of the IT). The generally conserved morphometric traits among the sister groups of populations might reflect the ecological similarities between populations separated by lowland barriers.

## Conclusions

Mitochondrial and nuclear DNA datasets show that the amethyst‐throated hummingbird is composed of two main lineages separated by the IT, congruent with previous finding based on a much smaller sample of mtDNA sequences (Cortés‐Rodríguez et al. 2008). Further within the group west of the IT, our data revealed three distinct evolutionary lineages (SMO, TMVB, and SMS), whereas east of the IT we do not have necessary sampling density to identify the different evolutionary lineages, though the data suggest at least two groups (north of MJP and along the MJP fault system). Species divergence time estimates and species distribution modeling provide evidence that the Pleistocene climatic cycles played an important role in isolating and limiting gene flow between the identified groups of populations. In addition, demographic reconstructions indicate no significant demographic expansions. These results suggest that groups of populations remained in situ primarily within the currently fragmented distribution of the cloud forest, and that the timing of intraspecific divergence events followed a postglacial divergence scenario. Combining the genetic data with differences in morphometric traits and plumage coloration support the recognition of the blue‐throated populations from Guerrero (*L. amethystinus margaritae*) as a separate evolutionary unit, with the assignment of the previously unknown blue‐throated populations from Sierra de Miahuatlán, Oaxaca to *L. margaritae* instead of *L. amethystinus circumventus*. Tests of divergence scenarios using multilocus ABC allowed us to reject hypotheses of geographic isolation as a driver for *L. amethystinus* speciation, but supported a role of refugial‐like dynamics during Pleistocene glacial cycles in generating recent intraspecific genetic structure. We confirmed the importance of geographic barriers such as the Isthmus of Tehuantepec, stressing the need to investigate cryptic barriers and not only conspicuous landscape features such as the IT. Overall, our data support the idea of multiple and varied mechanisms contributing to phenotypic and genetic differentiation of this member of the Mesoamerican biota. The pattern of genetic diversity that we have described herein is likely to be part of a larger phylogeographic signature that was retained as co‐distributed species of the associated cloud forest biota experienced a common history of vicariant events and climatic changes. Understanding the comparative phylogeography of such groups will aid the development and evaluation of phylogeographical hypotheses about the evolutionary history of the Mesoamerican biota and have implications for the conservation of cryptic species.

## Conflict of Interest

None declared.

## Data Accessibility

DNA sequences: GenBank Accessions numbers KU375264–KU375338, KU375339–KU375423. Specimen information: uploaded as Table S1.

Sequence alignments, microsatellite data, and input files (IMa, MAXENT, DIYABC) from the Dryad Digital Repository : http://dx.doi.org/10.5061/dryad.88589.

## Supporting information


**Figure S1.** Map of the collection sites of *Lampornis amethystinus*.

**Figure S2.** Bayesian skyline plots showing means for the historical demographic trends of *Lampornis amethystinus* groups using mitochondrial sequences.

**Figure S3.** Chronogram based on a Bayesian approach using a coalescent tree prior under and assuming constant population size of *Lampornis amethystinus* mtDNA sequences in BEAST.

**Figure S4.** (A) Competing demographic scenarios of *Lampornis amethystinus* divergence and admixture: *isolation split model 1* (scenario 1, left) predicts that TMVB (Pop1) merged with SMO (Pop2) at t1 then SMO merged with SMS (Pop3, *margaritae*) at t2 and subsequently with CHIS east of IT (Pop4) at t3; *isolation split model 2* (scenario 2, centre) is similar to the previous one but predicts that SMS (Pop3) merged with TMVB (Pop1) at t1 then TMVB merged with SMO (Pop2) at t2 and subsequently with CHIS east of IT (Pop4) at t3; *isolation with admixture model* (scenario 3, right) consisted of the same basal split between CHIS (Pop4) and the rest of groups west of IT described in previous scenarios but includes a hybridization/lineage fusion event in which SMS (Pop3) is the descendent of admixture between TMVB (Pop1) and SMO (Pop2) at t1, then Pop1 merged with Pop2 at t2, and subsequently with Pop4 at t3.

**Figure S5.** Results from the MAXENT analyses showing species distribution models for *Lampornis amethystinus* at (a) Last Interglacial (LIG, 140–120 ka), (b) Last Glacial Maximum (LGM, CCSM, 21 ka), (c) Last Glacial Maximum (LGM, MIROC, 21 ka), and (d) at present.

**Figure S6.** Morphological differences between groups of populations of *Lampornis amethystinus (*males, A–C; females, D–E).

**Table S1.** Collection localities for the *Lampornis amethystinus* samples examined here.

**Table S2.** Number of genetically analysed samples (*n*) for the combined *ND2* and cytochrome *b* sequences of *Lampornis amethystinus*, number of distinct haplotypes (H) found in individuals sampled for each mitochondrial fragment, and the number of individuals per haplotype in parentheses.

**Table S3.** Pairwise comparisons of *F*_*ST*_ values of mtDNA (above the diagonal) and microsatellites (below the diagonal) among populations of *Lampornis amethystinus* grouped based on mountain geography.

**Table S4.** Population genetic variability of groups of populations of *Lampornis amethystinus*.

**Table S5.** Number of genetically analysed samples for eight microsatellites (*n*), mean alleles per locus, observed (*H*_*O*_) and expected heterozygosity (*H*_*E*_), and significant deviations of Hardy–Weinberg equilibrium (HWE) for groups of populations of *Lampornis amethystinus*.

**Table S6.** Model parameters estimated from prior distributions of Scenario 1 (TMVB merged with SMO at t1, then SMO merged with SMS at t2 and subsequently with CHIS population at t3) using Approximate Bayesian Computation (ABC).

**Table S7.** Comparison of summary statistics for the observed data set and posterior simulated data sets of microsatellites and mitochondrial DNA sequences for *Lampornis amethystinus*.
