# Supplementary figures and images for: Molecular and iridescent feather reflectance data reveal recent genetic diversification and phenotypic differentiation in a cloud forest hummingbird
Source: Ecol Evol. 2016 Jan 22;6(4):1104–27. doi: 10.1002/ece3.1950 (PMC4722824; doi:10.1002/ece3.1950)

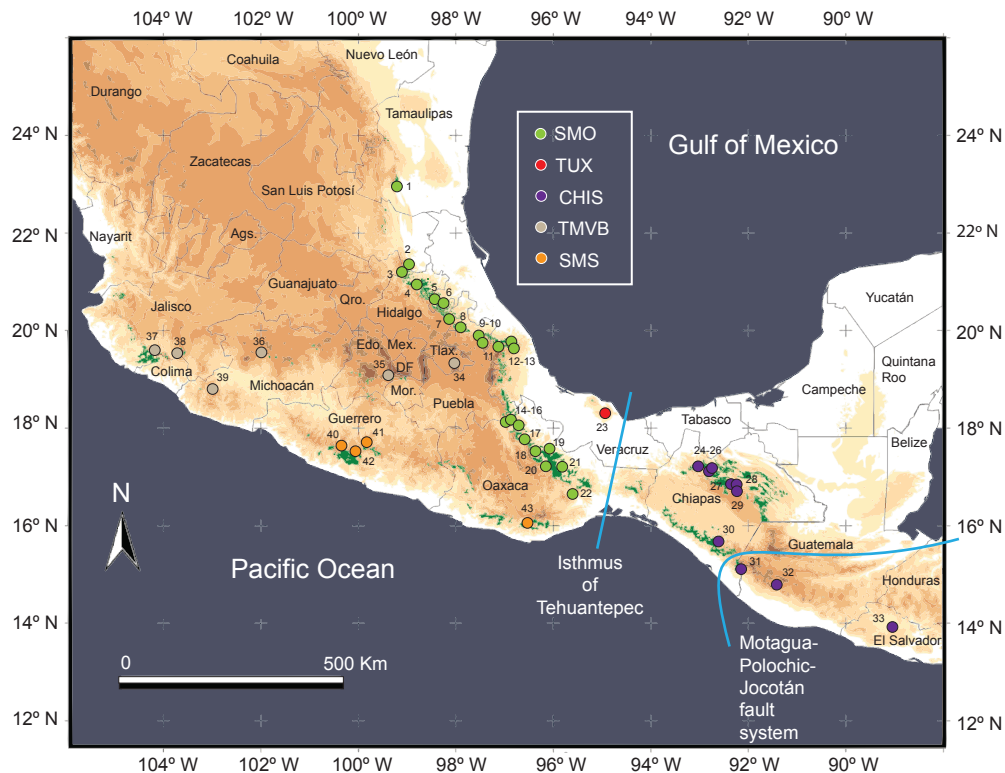

Supplement: Supplementary file 1 — Figure S1. Map of the collection sites of Lampornis amethystinus. [file ECE3-6-1104-s001.pdf]

Effective population size \* generation time

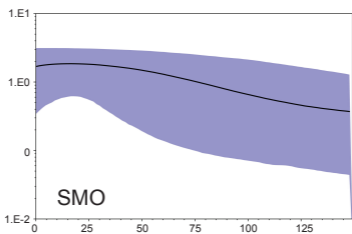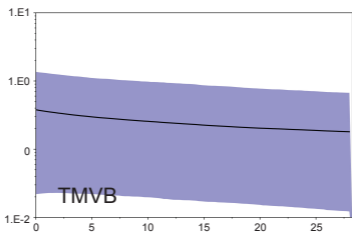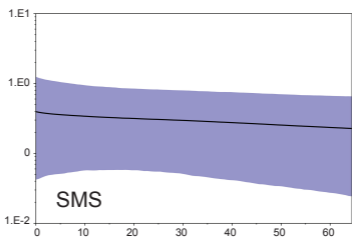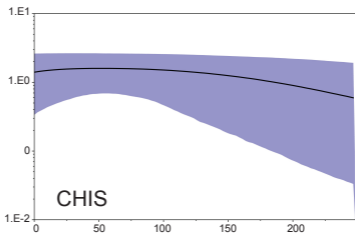

Time (thousand years ago)

Supplement: Supplementary file 2 — Figure S2. Bayesian skyline plots showing means for the historical demographic trends of Lampornis amethystinus groups using mitochondrial sequences. [file ECE3-6-1104-s002.pdf]

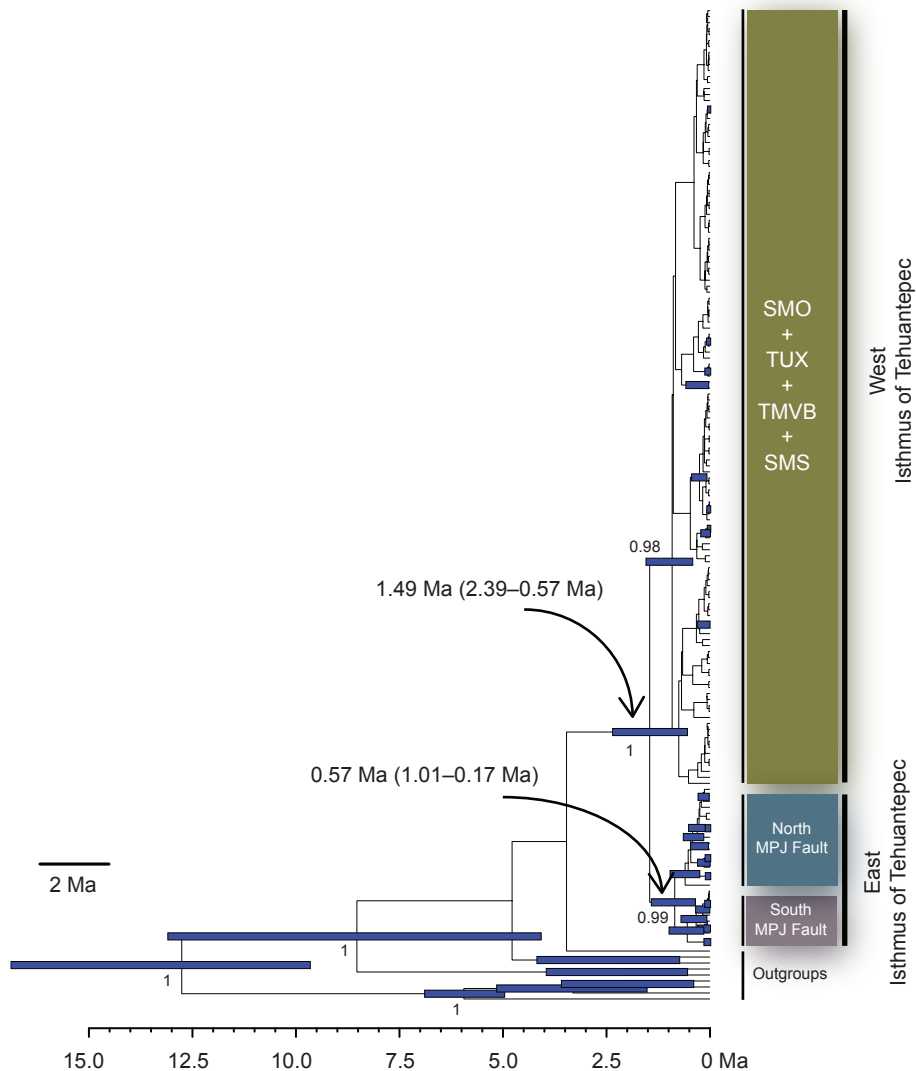

Supplement: Supplementary file 3 — Figure S3. Chronogram based on a Bayesian approach using a coalescent tree prior under and assuming constant population size of Lampornis amethystinus mtDNA sequences in BEAST. [file ECE3-6-1104-s003.pdf]

(A)

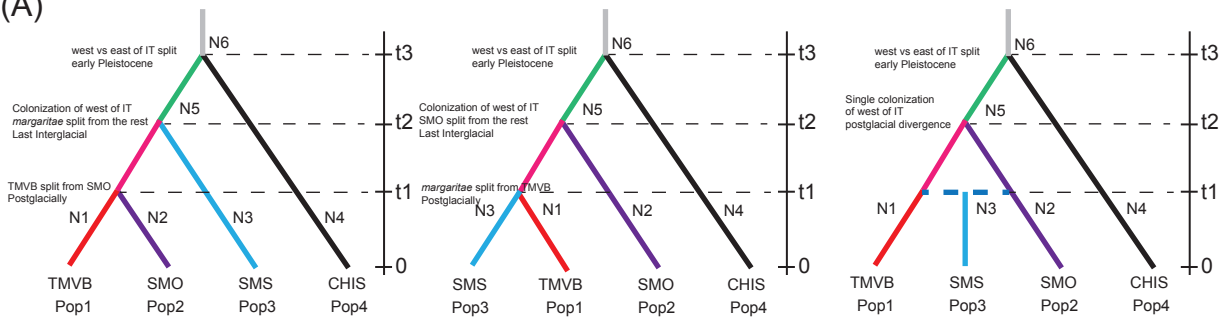

(B)

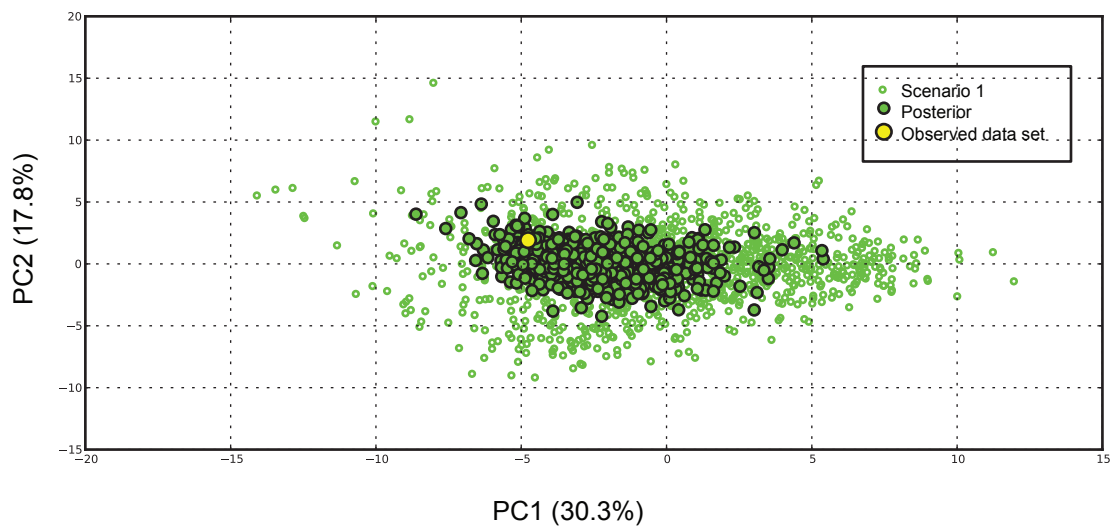

(C)

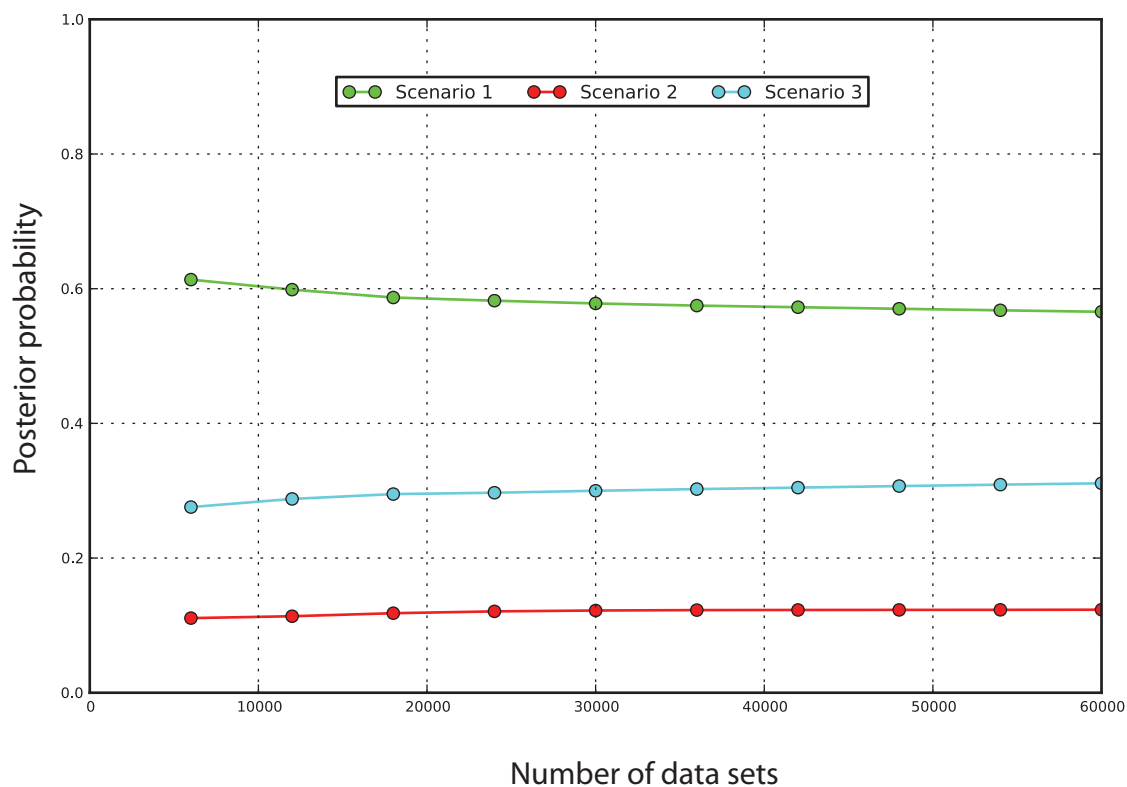

Supplement: Supplementary file 4 — Figure S4. (A) Competing demographic scenarios of Lampornis amethystinus divergence and admixture: isolation split model 1 (scenario 1, left) predicts that TMVB (Pop1) merged with SMO (Pop2) at t1 then SMO merged with SMS (Pop3, margaritae) at t2 and subsequently with CHIS east of IT (Pop4) at t3; isolation split model 2 (scenario 2, centre) is similar to the previous one but predicts that SMS (Pop3) merged with TMVB (Pop1) at t1 then TMVB merged with SMO (Pop2) at t2 and subsequently with CHIS east of IT (Pop4) at t3; isolation with admixture model (scenario 3, right) consisted of the same basal split between CHIS (Pop4) and the rest of groups west of IT described in previous scenarios but includes a hybridization/lineage fusion event in which SMS (Pop3) is the descendent of admixture between TMVB (Pop1) and SMO (Pop2) at t1, then Pop1 merged with Pop2 at t2, and subsequently with Pop4 at t3. [file ECE3-6-1104-s004.pdf]

LIG

LGM (CCSM)

LGM (MIROC)

Present

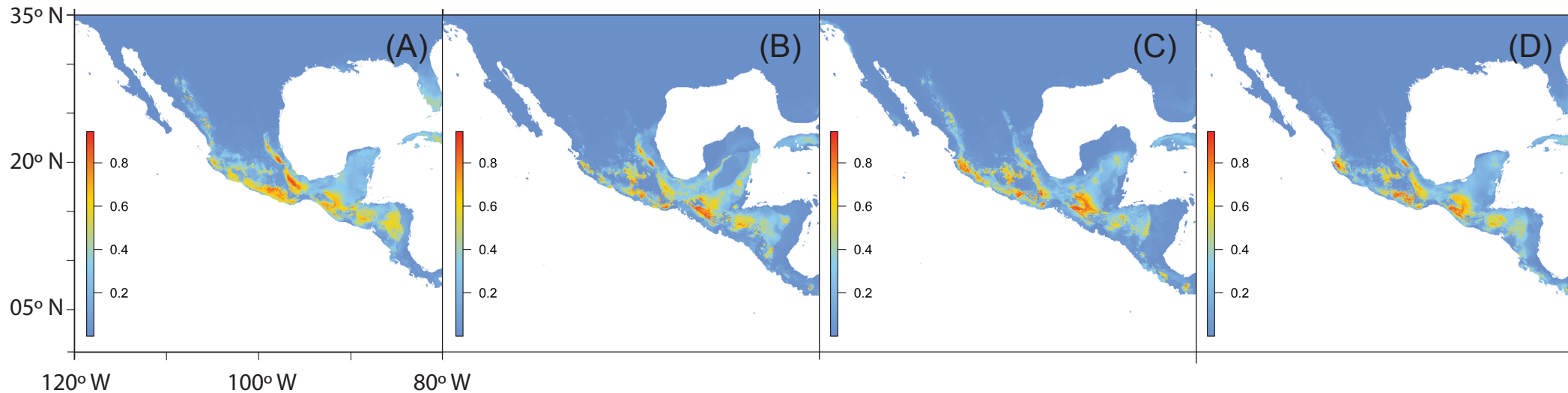

Supplement: Supplementary file 5 — Figure S5. Results from the MAXENT analyses showing species distribution models for Lampornis amethystinus at (a) Last Interglacial (LIG, 140–120 ka), (b) Last Glacial Maximum (LGM, CCSM, 21 ka), (c) Last Glacial Maximum (LGM, MIROC, 21 ka), and (d) at present. [file ECE3-6-1104-s005.pdf]

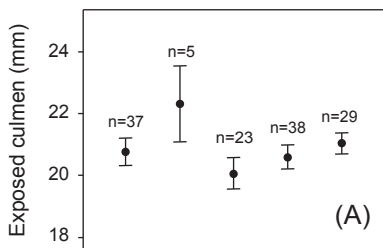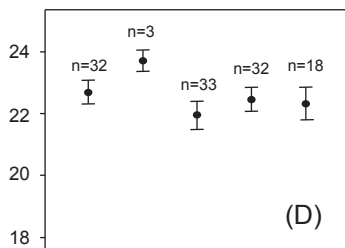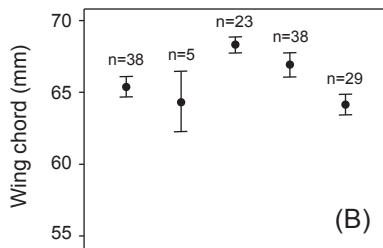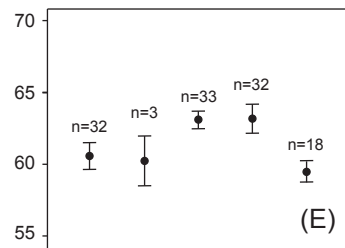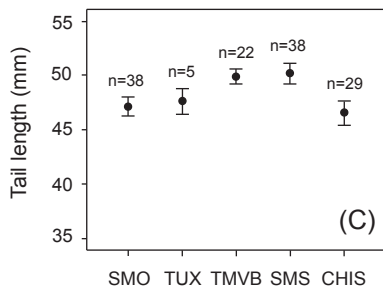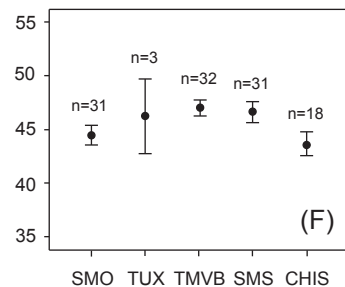

Supplement: Supplementary file 6 — Figure S6. Morphological differences between groups of populations of Lampornis amethystinus (males, A–C; females, D–E). [file ECE3-6-1104-s006.pdf]
