# Supplementary material for: Molecular and iridescent feather reflectance data reveal recent genetic diversification and phenotypic differentiation in a cloud forest hummingbird
Source: Ecol Evol. 2016 Jan 22;6(4):1104–27. doi: 10.1002/ece3.1950 (PMC4722824; doi:10.1002/ece3.1950)
Supplement: Supplementary file 7 — Table S1. Collection localities for the Lampornis amethystinus samples examined here. [file ECE3-6-1104-s007.doc]

**Table S1**. Collection localities for the *Lampornis amethystinus* samples examined here. mtDNA = sample size for mitochondrial DNA sequences, SSRs = sample size for nuclear microsatellites.

| Location Code | Location | Region§ | mtDNA§§ | SSRs | Altitude  (m asl) | Latitude  (N) | Longitude (W) |
| --- | --- | --- | --- | --- | --- | --- | --- |
|  |  |  |  |  |  |  |  |
| 1 | Tamaulipas, El Cielo | SMO | 2 | 0 | 943 | 23º 03´ 33´´ | 99º 12´ 21´´ |
| 2 | San Luis Potosí, Xilitla | SMO | 2 | 2 | 637 | 21° 22´ 39´´ | 98° 59´ 35´´ |
| 3 | Querétaro, El Pemoche | SMO | (2) | 2 | 1441 | 21º 13´ 34´´ | 99º 06´ 34´´ |
| 4 | Hidalgo, Tlanchinol | SMO | 8 (6) | 4 | 1476 | 21º 01´ 24´´ | 98º 36´ 37´´ |
| 5 | Veracruz, Huayacocotla | SMO | 3 | 3 | 1397 | 20º 37´ 18´´ | 98º 27´ 50´´ |
| 6 | Veracruz, Zacualpan | SMO | (5) | 5 | 1772 | 20º 28´ 01´´ | 98º 18´ 52´´ |
| 7 | Hidalgo, Tenango de Doria | SMO | (5) | 0 | 1431 | 20º 19´ 10´´ | 98º 13´ 18´´ |
| 8 | Puebla, Lagunillas | SMO | 2 | 2 | 1500 | 20º 13´ 49´´ | 97º 57´ 19´´ |
| 9 | Puebla, La Galera | SMO | 6 | 6 | 950 | 19º 59´ 10´´ | 97º 36´ 35´´ |
| 10 | Puebla, Huitzilan | SMO | 1 | 1 | 975 | 19º 57´ 29´´ | 97º 41´ 17´´ |
| 11 | Puebla, Teziutlán | SMO | 3 | 2 | 1910 | 19º 49´ 39´´ | 97º 20´ 10´´ |
| 12 | Veracruz, Clavijero | SMO | 1 | 0 | 1225 | 19º 30´ 47´´ | 96º 56´ 28´´ |
| 13 | Veracruz, El Riscal | SMO | 6 | 5 | 1586 | 19º 28´ 22´´ | 96º 59´ 51´´ |
| 14 | Oaxaca, Puerto de la Soledad | SMO | (3) | 2 | 2334 | 18º 09´ 57´´ | 96º 59´ 52´´ |
| 15 | Oaxaca, Santa Ana Ateixtlahuaca | SMO | 5 | 5 | 1830 | 18º 12´ 18´´ | 96º 54´ 00´´ |
| 16 | Oaxaca, San Pedro Ocopetatillo | SMO | 2 | 2 | 1652 | 18º 11´ 05´´ | 96º 54´ 31´´ |
| 17 | Oaxaca, San Martín Caballero | SMO | (4) | 2 | 1403 | 18º 06´ 41´´ | 96º 38´ 24´´ |
| 18 | Oaxaca, Peña Verde | SMO | (2) | 3 | 1717 | 17º 50´ 42´´ | 96º 44´ 24´´ |
| 19 | Oaxaca, Valle Nacional | SMO | 2 | 1 | 1198 | 17º 39´ 16´´ | 96º 20´ 13´´ |
| 20 | Oaxaca, Santiago Comaltepec | SMO | 1 | 1 | 2205 | 17º 33´ 55´´ | 96º 32´ 10´´ |
| 21 | Oaxaca, Cerro de Zempoaltepec | SMO | (2) | 0 | 975 | 17º 25´ 28´´ | 95º 58´ 59´´ |
| 22 | Oaxaca, Cerro Piedra Larga | SMO | (3) | 0 | 1349 | 16º 09´ 40´´ | 97º 00´ 37´´ |
| 23 | Veracruz, Sierra de Santa Marta | TUX | (4) | 0 | 1613 | 18º 20´ 45´´ | 94º 51´ 28´´ |
| 24 | Chiapas, Pueblo Nuevo | CHIS | 6 (3) | 6 | 1639 | 17º 08´ 54´´ | 92º 50´ 58´´ |
| 25 | Chiapas, Jitotol | CHIS | 4 | 4 | 1692 | 17º 01´ 47´´ | 92º 50´ 46´´ |
| 26 | Chiapas, Rancho Nuevo | CHIS | 2 | 3 | 1640 | 17º 08´ 00´´ | 92º 45´ 24´´ |
| 27 | Chiapas, Huitepec | CHIS | (3) | 0 | 2386 | 16º 49´ 37´´ | 92º 35´ 13´´ |
| 28 | Chiapas, San Cristobal de las Casas | CHIS | (2) | 0 | 2189 | 16º 49´ 00´´ | 92º 35´ 00´´ |
| 29 | Chiapas, Cerro Tultepec | CHIS | 0 | 11 | 2422 | 16º 45´ 25´´ | 92º 39´ 31´´ |
| 30 | Chiapas, El Triunfo | CHIS | (1) | 0 | 1032 | 15º 37´ 00´´ | 92º 50´ 00´´ |
| 31 | Chiapas, Volcán Tacaná | CHIS | 6 (2) | 5 | 1753 | 15º 05´ 34´´ | 92º 05´ 31´´ |
| 32 | Guatemala, S. de las Minas, Quetzaltenango | CHIS | (2) | 0 | 2366 | 14º 46´ 40´´ | 91º 40´ 53´´ |
| 33 | El Salvador, Chalatenango, Cerro El Pital | CHIS | (1) | 0 | 629 | 14º 01´ 51´´ | 88º 52´ 33´´ |
| 34 | Tlaxcala, La Malinche | TMVB | 1 | 1 | 2900 | 19º 14´ 41´´ | 98º 06´ 58´´ |
| 35 | Edo. de México, Ocuilan de Arteaga | TMVB | (4) | 3 | 2166 | 18º 56´ 42´´ | 99º 15´ 53´´ |
| 36 | Michoacán, Zirimondiro | TMVB | (2) | 1 | 2246 | 19º 36´ 00´´ | 102º 20´ 17´´ |
| 37 | Jalisco, Sierra de Manantlán | TMVB | 4 | 0 | 1990 | 19º 31´ 42´´ | 104º 10´ 46´´ |
| 38 | Jalisco, Nevado de Colima | TMVB | 5 | 5 | 2182 | 19º 31´ 42´´ | 103º 37´ 00´´ |
| 39 | Michoacán, Coalcomán | TMVB | (3) | 0 | 1058 | 18º 48´ 24´´ | 102º 57´ 05´´ |
| 40 | Guerrero, El Iris | SMS | (6) | 0 | 2319 | 17º 29´ 00´´ | 100º 12´ 00´´ |
| 41 | Guerrero, Omiltemi | SMS | 13 | 13 | 2900 | 17º 53´ 03´´ | 99º 60´ 00´´ |
| 42 | Guerrero, Carrizal de Bravo | SMS | 8 (4) | 9 | 2373 | 17º 36´ 00´´ | 99º 50´ 00´´ |
| 43 | Oaxaca, Sierra de Miahuatlán | SMS | 10 | 10 | 2316 | 16º 05´ 20´´ | 96º 28´ 47´´ |
|  |  |  |  |  |  |  |  |

§ Region abbreviations are as follows: SMO = Sierra Madre Oriental; TUX = Sierra de Los Tuxtlas and Sierra de Santa Marta; SMS = Sierra Madre del Sur (Sierra de Miahuatlán, Oaxaca and Guerrero); TMVB = Trans-Mexican Volcanic Belt; CHIS = Chiapan Highlands separated by the Central Depression that together with Guatemala and El Salvador form the region TIH (Trans-Isthmian Highlands).

§§ Numbers in parentheses indicate samples in Cortés-Rodríguez et al. (2008).
