# Supplementary material for: Molecular and iridescent feather reflectance data reveal recent genetic diversification and phenotypic differentiation in a cloud forest hummingbird
Source: Ecol Evol. 2016 Jan 22;6(4):1104–27. doi: 10.1002/ece3.1950 (PMC4722824; doi:10.1002/ece3.1950)
Supplement: Supplementary file 8 — Table S2. Number of genetically analysed samples (n) for the combined ND2 and cytochrome b sequences of Lampornis amethystinus, number of distinct haplotypes (H) found in individuals sampled for each mitochondrial fragment, and the number of individuals per haplotype in parentheses. [file ECE3-6-1104-s008.doc]

**Table S2**. The number of genetically analysed samples (*n*) for mitochondrial DNA sequences (combined *ND2* and cyt *b*) and the number of distinct haplotypes (H) found in the *Lampornis* *amethystinus* hummingbirds sampled, with the number of birds per haplotype in parentheses. Codes are from the network in Fig 1.

| Location Code | Location | Region§ | *n* | *ND2* and cyt *b* haplotype |
| --- | --- | --- | --- | --- |
|  |  |  |  |  |
| 1 | Tamaulipas, El Cielo | SMO | 2 | H1(1), H2(1) |
| 2 | San Luis Potosí, Xilitla | SMO | 2 | H3(1), H4(1) |
| 3 | Querétaro, El Pemoche | SMO | 2 | H1(1), H5(1) |
| 4 | Hidalgo, Tlanchinol | SMO | 8 | H1(1), H5(2), H6(1), H7(1), H8(1), H9(1), H10(1) |
| 5 | Veracruz, Huayacocotla | SMO | 3 | H4(1), H5(2) |
| 6 | Veracruz, Zacualpan | SMO | 5 | H1(1), H5(1), H13(1), H14(1), H15(1) |
| 7 | Hidalgo, Tenango de Doria | SMO | 5 | H2(2), H5(1), H11(1), H12(1) |
| 8 | Puebla, Lagunillas | SMO | 2 | H5(1), H17(1) |
| 9 | Puebla, La Galera | SMO | 6 | H4(1), H5(2), H6(1), H16(2) |
| 10 | Puebla, Huitzilan | SMO | 1 | H16(1) |
| 11 | Puebla, Teziutlán | SMO | 3 | H5(1), H16(2) |
| 12 | Veracruz, Clavijero | SMO | 1 | H18(1) |
| 13 | Veracruz, El Riscal | SMO | 6 | H5(1), H6(1), H16(1), H18(1), H19(1), H20(1) |
| 14 | Oaxaca, Puerto de la Soledad | SMO | 3 | H2(1), H5(2) |
| 15 | Oaxaca, Santa Ana Ateixtlahuaca | SMO | 5 | H5(1), H16(3), H18(1) |
| 16 | Oaxaca, San Pedro Ocopetatillo | SMO | 2 | H18(2) |
| 17 | Oaxaca, San Martín Caballero | SMO | 4 | H1(1), H2(2), H23(1) |
| 18 | Oaxaca, Peña Verde | SMO | 2 | H24(1), H25(1) |
| 19 | Oaxaca, Valle Nacional | SMO | 2 | H21(1), H22(1) |
| 20 | Oaxaca, Santiago Comaltepec | SMO | 1 | H16(1) |
| 21 | Oaxaca, Cerro de Zempoaltepec | SMO | 2 | H1(1), H26(1) |
| 22 | Oaxaca, Cerro Piedra Larga | SMO | 3 | H1(3) |
| 23 | Veracruz, Sierra de Santa Marta | TUX | 4 | H2(3), H32(1) |
| 24 | Chiapas, Pueblo Nuevo | CHIS | 6 | H43(1), H44(1), H45(1), H46(1), H47(2) |
| 25 | Chiapas, Jitotol | CHIS | 4 | H52(1), H53(1), H54(1), H55(1) |
| 26 | Chiapas, Rancho Nuevo | CHIS | 2 | H56(1), H57(1) |
| 27 | Chiapas, Huitepec | CHIS | 3 | H50(1), H51(2) |
| 28 | Chiapas, San Cristobal de las Casas | CHIS | 2 | H48(1), H49(1) |
| 29 | Chiapas, Cerro Tultepec | CHIS | 0 | – |
| 30 | Chiapas, El Triunfo | CHIS | 1 | H58(1) |
| 31 | Chiapas, Volcán Tacaná | CHIS | 6 | H59(2), H60(1), H61(1), H62(2) |
| 32 | Guatemala, S. de las Minas, Quetzaltenango | CHIS | 2 | H59(2) |
| 33 | El Salvador, Chalatenango, Cerro El Pital | CHIS | 1 | H60(1) |
| 34 | Tlaxcala, La Malinche | TMVB | 1 | H5(1) |
| 35 | Estado de México, Ocuilan de Arteaga | TMVB | 4 | H1(4) |
| 36 | Michoacán, Zirimondiro | TMVB | 2 | H1(1), H33(1) |
| 37 | Jalisco, Sierra de Manantlán | TMVB | 4 | H1(1) |
| 38 | Jalisco, Nevado de Colima | TMVB | 5 | H16(4), H34(1) |
| 39 | Michoacán, Coalcomán | TMVB | 3 | H1(3) |
| 40 | Guerrero, El Iris | SMS | 6 | H37(3), H42(3) |
| 41 | Guerrero, Omiltemi | SMS | 13 | H16(6), H35(1), H36(1), H37(4), H38(1) |
| 42 | Guerrero, Carrizal de Bravo | SMS | 8 | H1(1), H37(3), H39(1), H40(1), H41(2) |
| 43 | Oaxaca, Sierra de Miahuatlán | SMS | 10 | H16(4), H27(2), H28(1), H29(1), H30(1), H31(1) |
|  |  |  |  |  |

§ Region abbreviations are as follows: SMO = Sierra Madre Oriental; TUX = Sierra de Los Tuxtlas and Sierra de Santa Marta; SMS = Sierra Madre del Sur (Sierra de Miahuatlán, Oaxaca and Guerrero); TMVB = Trans-Mexican Volcanic Belt; CHIS = Chiapan Highlands separated by the Central Depression that together with Guatemala and El Salvador form the region TIH (Trans-Isthmian Highlands).
