# Supplementary material for: Molecular and iridescent feather reflectance data reveal recent genetic diversification and phenotypic differentiation in a cloud forest hummingbird
Source: Ecol Evol. 2016 Jan 22;6(4):1104–27. doi: 10.1002/ece3.1950 (PMC4722824; doi:10.1002/ece3.1950)
Supplement: Supplementary file 9 — Table S3. Pairwise comparisons of F ST values of mtDNA (above the diagonal) and microsatellites (below the diagonal) among populations of Lampornis amethystinus grouped based on mountain geography. [file ECE3-6-1104-s009.doc]

**Table S3.** Pairwise comparisons of *F_ST_* values of mtDNA (above the diagonal) and microsatellites (below the diagonal) among populations of *Lampornis amethystinus* grouped by mountain geography.

| Region | SMO | TUX | TMVB | SMS | CHIS |
| --- | --- | --- | --- | --- | --- |
| SMO | _ | 0.003 | **0.108** | **0.169** | **0.632** |
| TUX | _ | _ | 0.183 | 0.207 | **0.499** |
| TMVB | **0.055** | _ | _ | **0.108** | **0.600** |
| SMS | **0.029** | _ | **0.121** | _ | **0.627** |
| CHIS | **0.086** | _ | **0.135** | **0.070** | _ |

Significant values at *P* < 0.01 values after Bonferroni’s correction (*P* = 0.005) are shown in bold. Region abbreviations are as follows: SMO = Sierra Madre Oriental; TUX = Sierra de Los Tuxtlas and Sierra de Santa Marta; SMS = Sierra Madre del Sur (Sierra de Miahuatlán, Oaxaca and Guerrero); TMVB = Trans-Mexican Volcanic Belt; CHIS = Chiapan Highlands separated by the Central Depression that together with Guatemala and El Salvador form the region TIH (Trans-Isthmian Highlands). TUX was not included in microsatellite pairwise comparisons across groups because small sample size.
