# Supplementary material for: Molecular and iridescent feather reflectance data reveal recent genetic diversification and phenotypic differentiation in a cloud forest hummingbird
Source: Ecol Evol. 2016 Jan 22;6(4):1104–27. doi: 10.1002/ece3.1950 (PMC4722824; doi:10.1002/ece3.1950)
Supplement: Supplementary file 10 — Table S4. Population genetic variability of groups of populations of Lampornis amethystinus. [file ECE3-6-1104-s010.doc]

**Table S4.** Population genetic variability of groups of populations of *Lampornis amethystinus*. Number of genetically analysed samples for eight microsatellites, mean alleles per locus, allelic richness, and observed (*H_O_*) and expected heterozygosity (*H_E_*). Data are means ± SD.

| Region | *n* | Mean alleles/  locus | Allelic richness | *H_O_* | *H_E_* |
| --- | --- | --- | --- | --- | --- |
| SMO | 48 | 7.25 ± 3.8 | 4.69 ± 2.4 | 0.49 ± 0.24 | 0.61 ± 0.27*✝ |
| TMVB | 10 | 5.40 ± 1.1 | 5.18 ± 2.5 | 0.56 ± 0.36 | 0.76 ± 0.08* |
| SMS | 32 | 7.43 ± 2.9 | 4.75 ± 2.3 | 0.53 ± 0.23 | 0.62 ± 0.25*✝§ |
| CHIS | 29 | 5.88 ± 2.7 | 5.39 ± 2.7 | 0.47 ± 0.31 | 0.59 ± 0.34*✝ |

Asterisks indicate a significant departure for HumB15 (*), CACU16-1 (✝) and HumB9 (§) from Hardy-Weinberg equilibrium after a sequential Bonferroni correction (*P* < 0.0006). Region abbreviations are as follows: SMO = Sierra Madre Oriental; TUX = Sierra de Los Tuxtlas and Sierra de Santa Marta; TMVB = Trans-Mexican Volcanic Belt; MIA = Sierra de Miahuatlán, Oaxaca; SMS = Sierra Madre del Sur (Guerrero); CHIS = Chiapan Highlands separated by the Central Depression that together with Guatemala and El Salvador form the region TIH (Trans-Isthmian Highlands). Hummingbirds from TUX population were not genotyped.
