# Supplementary material for: Molecular and iridescent feather reflectance data reveal recent genetic diversification and phenotypic differentiation in a cloud forest hummingbird
Source: Ecol Evol. 2016 Jan 22;6(4):1104–27. doi: 10.1002/ece3.1950 (PMC4722824; doi:10.1002/ece3.1950)
Supplement: Supplementary file 11 — Table S5. Number of genetically analysed samples for eight microsatellites (n), mean alleles per locus, observed (H O) and expected heterozygosity (H E), and significant deviations of Hardy–Weinberg equilibrium (HWE) for groups of populations of Lampornis amethystinus. [file ECE3-6-1104-s011.doc]

**Table S5**. Number of genetically analysed samples for eight microsatellites (*n*), mean alleles per locus, total number of alleles, observed (*H_O_*) and expected heterozygosity (*H_E_*), and significant deviations of Hardy–Weinberg equilibrium (HWE) for groups of populations of *Lampornis amethystinus*.

| Locus | SMO | TMVB | SMS | CHIS | Mean/  alleles/locus | Total | *H*_O_ | *H*_E_ | HWE |
| --- | --- | --- | --- | --- | --- | --- | --- | --- | --- |
| CACU16-1 | 48 | 10 | 30 | 28 | 9 ± 3.16 | 15 | 0.58 ± 0.03 | 0.83 ± 0.05 | *P* < 0.006 |
| CACU17-2 | 48 | 10 | 28 | 28 | 6.75 ± 2.5 | 11 | 0.59 ± 0.22 | 0.68 ± 0.05 | n.s. |
| HumB15 | 45 | 10 | 29 | 27 | 8.25 ± 1.7 | 8 | 0.30 ± 0.15 | 0.83 ± 0.03 | *P* < 0.006 |
| HumB2 | 48 | 10 | 31 | 29 | 4.5 ± 2.89 | 8 | 0.51 ± 0.32 | 0.48 ± 0.21 | n.s. |
| HumB9 | 48 | 10 | 31 | 29 | 5.5 ± 1.0 | 4 | 0.63 ± 0.25 | 0.70 ± 0.08 | *P* < 0.006 |
| HumB10 | 47 | 10 | 28 | 29 | 1.75 ± 0.96 | 3 | 0.10 ± 0.09 | 0.01 ± 0.10 | n.s. |
| HumB11 | 48 | 10 | 29 | 26 | 2.25 ± 0.96 | 14 | 0.22 ± 0.19 | 0.21 ± 0.19 | n.s. |
| HumB3 | 48 | 10 | 32 | 29 | 9 ± 2.16 | 11 | 0.83 ± 0.14 | 0.82 ± 0.08 | n.s. |

Region abbreviations are as follows: SMO = Sierra Madre Oriental; SMS = Sierra Madre del Sur (Sierra de Miahuatlán, Oaxaca and Guerrero); TMVB = Trans-Mexican Volcanic Belt; CHIS = Chiapan Highlands separated by the Central Depression that together with Guatemala and El Salvador form the region TIH (Trans-Isthmian Highlands); TUX was not included in pairwise comparisons across groups because small sample size. HWE = *P* values of the exact test of Hardy–Weinberg equilibrium.
