# Supplementary material for: Molecular and iridescent feather reflectance data reveal recent genetic diversification and phenotypic differentiation in a cloud forest hummingbird
Source: Ecol Evol. 2016 Jan 22;6(4):1104–27. doi: 10.1002/ece3.1950 (PMC4722824; doi:10.1002/ece3.1950)
Supplement: Supplementary file 12 — Table S6. Model parameters estimated from prior distributions of Scenario 1 (TMVB merged with SMO at t1, then SMO merged with SMS at t2 and subsequently with CHIS population at t3) using Approximate Bayesian Computation (ABC). [file ECE3-6-1104-s012.doc]

**Table S6.** Posterior parameter estimates (mean, median and 90–95% confidence intervals) for the best-supported scenario (scenario 1) considering the four amethyst-throated hummingbird groups (TMVB, SMO, SMS and CHIS). Estimates are based on 1% of simulated datasets closest to the observed values. Simulations and approximate Bayesian computation (ABC) analyses were performed considering both mtDNA and microsatellites.

| Parameters | Mean | Median | Quantile 2.5% | Quantile 5% | Quantile 95% | Quantile 97.5% |
| --- | --- | --- | --- | --- | --- | --- |
|  |  |  |  |  |  |  |
| *N*TMVB | 6.14 × 10^3^ | 5.12 × 10^3^ | 1.23 × 10^3^ | 1.58 × 10^3^ | 1.47 × 10^4^ | 1.70 × 10^4^ |
| *N*SMO | 1.75 × 10^4^ | 1.75 × 10^4^ | 6.88 × 10^3^ | 8.26 × 10^3^ | 2.70 × 10^4^ | 2.85 × 10^4^ |
| *N*SMS | 1.36 × 10^4^ | 1.38 × 10^4^ | 6.44 × 10^3^ | 7.71 × 10^3^ | 1.86 × 10^4^ | 1.93 × 10^4^ |
| *N*CHIS | 1.92 × 10^4^ | 1.83 × 10^4^ | 5.93 × 10^3^ | 7.43 × 10^3^ | 3.46 × 10^4^ | 3.70 × 10^4^ |
| *N5* | 2.94 × 10^4^ | 2.97 × 10^4^ | 8.66 × 10^3^ | 1.15 × 10^4^ | 4.59 × 10^4^ | 4.78 × 10^4^ |
| *N6* | 2.84 × 10^4^ | 2.13 × 10^4^ | 5.59 × 10^3^ | 6.23 × 10^3^ | 7.58 × 10^4^ | 8.60 × 10^4^ |
| *t*1 | 4.40 × 10^2^ | 3.39 × 10^2^ | 1.34 × 10^2^ | 1.52 × 10^2^ | 1.06 × 10^3^ | 1.36 × 10^3^ |
| *t*2 | 1.10 × 10^3^ | 7.28 × 10^2^ | 2.12 × 10^2^ | 2.45 × 10^2^ | 3.42 × 10^3^ | 4.38 × 10^3^ |
| *t*3 | 5.43 × 10^3^ | 3.61 × 10^3^ | 7.93 × 10^2^ | 1.02 × 10^3^ | 1.66 × 10^4^ | 2.47 × 10^4^ |
| *μ*mtDNA | 1.87 × 10­–6 | 1.71 × 10^–6^ | 9.11 × 10^–7^ | 1.01 × 10^–6^ | 3.22 × 10^–6^ | 3.79 × 10^–6^ |
| *μ*micros | 6.20 × 10^–5^ | 5.57 × 10^–5^ | 2.08 × 10^–5^ | 2.51 × 10^–5^ | 1.20 × 10^–4^ | 1.38 × 10^–4^ |
|  |  |  |  |  |  |  |

Parameters are *N* = effective population size for TMVB (pop1, N1), SMO (Pop2, N2), SMS (Pop3, N3), CHIS (Pop4, N4) and the ancestral populations (N5 and N6), *t* = time since divergence after the Last Glacial Maximum (t1), during Last Interglacial (t2) and early in the Pleistocene (t3), and *μ* = mutation rate for mitochondrial DNA and microsatellites, respectively. Region abbreviations are as follows: SMO = Sierra Madre Oriental; TUX = Sierra de Los Tuxtlas and Sierra de Santa Marta; SMS = Sierra Madre del Sur (Sierra de Miahuatlán, Oaxaca and Guerrero); TMVB = Trans-Mexican Volcanic Belt; CHIS = Chiapan Highlands separated by the Central Depression that together with Guatemala and El Salvador form the region TIH (Trans-Isthmian Highlands).
