# Supplementary material for: Molecular and iridescent feather reflectance data reveal recent genetic diversification and phenotypic differentiation in a cloud forest hummingbird
Source: Ecol Evol. 2016 Jan 22;6(4):1104–27. doi: 10.1002/ece3.1950 (PMC4722824; doi:10.1002/ece3.1950)
Supplement: Supplementary file 13 — Table S7. Comparison of summary statistics for the observed data set and posterior simulated data sets of microsatellites and mitochondrial DNA sequences for Lampornis amethystinus. [file ECE3-6-1104-s013.doc]

**Table S7.** Comparison of summary statistics for the observed data set and posterior simulated data sets for the *Lampornis* *amethystinus* best-fit scenario. Pop1 = Trans-Mexican Volcanic Belt (TMVB), Pop2 = Sierra Madre Oriental (SMO), Pop3 = Sierra Madre del Sur (SMS), Pop4 = Chiapan Highlands separated by the Central Depression that together with Guatemala and El Salvador form the Trans-Isthmian Highlands region (CHIS).

| Summary statistics | Observed value | *P*-value (simulated<observed) |
| --- | --- | --- |
| Mean number of alleles in Pop1 | 3.7500 | 0.0325* |
| Mean number of alleles in Pop2 | 7.3750 | 0.4330 |
| Mean number of alleles in Pop3 | 6.5000 | 0.3960 |
| Mean number of alleles in Pop4 | 5.8750 | 0.2805 |
| Mean genic diversity in Pop1 | 0.4757 | 0.0070** |
| Mean genic diversity in Pop2 | 0.6100 | 0.0335* |
| Mean genic diversity in Pop3 | 0.5434 | 0.0160* |
| Mean genic diversity in Pop4 | 0.5888 | 0.0520 |
| Mean size variance in Pop1 | 5.1678 | 0.5895 |
| Mean size variance in Pop2 | 6.2556 | 0.6700 |
| Mean size variance in Pop3 | 5.8518 | 0.6700 |
| Mean size variance in Pop4 | 5.8907 | 0.6560 |
| Mean genic diversity (Pop1 and Pop2) | 0.5990 | 0.0290* |
| Mean genic diversity (Pop1 and Pop3) | 0.5491 | 0.0150* |
| Mean genic diversity (Pop1 and Pop4) | 0.5887 | 0.0245* |
| Mean genic diversity (Pop2 and Pop3) | 0.5953 | 0.0250* |
| Mean genic diversity (Pop2 and Pop4) | 0.6262 | 0.0310* |
| Mean genic diversity (Pop3 and Pop4) | 0.5855 | 0.0150* |
| F_ST_ (Pop1 and Pop2) | 0.0542 | 0.9275 |
| F_ST_ (Pop1 and Pop3) | 0.0952 | 0.9655* |
| F_ST_ (Pop1 and Pop4) | 0.1090 | 0.7260 |
| F_ST_ (Pop2 and Pop3) | 0.0308 | 0.7045 |
| F_ST_ (Pop2 and Pop4) | 0.0733 | 0.5610 |
| F_ST_ (Pop3 and Pop4) | 0.0639 | 0.3910 |
| Number of haplotypes in Pop1 | 6 | 0.0590 |
| Number of haplotypes in Pop2 | 26 | 0.0960 |
| Number of haplotypes in Pop3 | 17 | 0.1440 |
| Number of haplotypes in Pop4 | 20 | 0.7795 |
| Number of segregating sites in Pop1 | 405 | 1.0000*** |
| Number of segregating sites in Pop2 | 39 | 0.0050** |
| Number of segregating sites in Pop3 | 16 | 0.0000*** |
| Number of segregating sites in Pop4 | 34 | 0.0385* |
| Mean pairwise differences in Pop1 | 141.9298 | 0.9980** |
| Mean pairwise differences in Pop2 | 2.9335 | 0.0000*** |
| Mean pairwise differences in Pop3 | 2.7057 | 0.0000*** |
| Mean pairwise differences in Pop4 | 5.4985 | 0.0140* |
| Number of haplotypes (Pop1 and Pop2) | 29 | 0.0270* |
| Number of haplotypes (Pop1 and Pop3) | 21 | 0.0185* |
| Number of haplotypes (Pop1 and Pop4) | 26 | 0.3165 |
| Number of haplotypes (Pop2 and Pop3) | 41 | 0.0560 |
| Number of haplotypes (Pop2 and Pop4) | 46 | 0.2210 |
| Number of haplotypes (Pop3 and Pop4) | 37 | 0.3850 |
| N_ST_ (Pop1 and Pop2) | 0.8571 | 1.0000*** |
| N_ST_ (Pop1 and Pop3) | 0.6407 | 0.9910** |
| N_ST_ (Pop1 and Pop4) | 0.4328 | 0.7705 |
| N_ST_ (Pop2 and Pop3) | 0.1694 | 0.8220 |
| N_ST_ (Pop2 and Pop4) | 0.6681 | 0.9615* |
| N_ST_ (Pop3 and Pop4) | 0.6436 | 0.9550* |
